# Electron Manipulation and Surface Reconstruction of Bimetallic Iron–Nickel Phosphide Nanotubes for Enhanced Alkaline Water Electrolysis

**DOI:** 10.1002/advs.202401207

**Published:** 2024-05-05

**Authors:** Xinqiang Wang, Jinhao Zhou, Wengang Cui, Fan Gao, Yong Gao, Fulai Qi, Yanxia Liu, Xiaoying Yang, Ke Wang, Zhenglong Li, Yaxiong Yang, Jian Chen, Wenping Sun, Lixian Sun, Hongge Pan

**Affiliations:** ^1^ Institute of Science and Technology for New Energy Xi'an Technological University Xi'an 710021 P. R. China; ^2^ Guangdong‐Hong Kong‐Macao Joint Laboratory for Intelligent Micro‐Nano Optoelectronic Technology School of Physics and Optoelectronic Engineering Foshan University Foshan 528225 P. R. China; ^3^ School of Materials Science and Engineering State Key Laboratory of Clean Energy Utilization Zhejiang University Hangzhou 310027 P. R. China; ^4^ School of Material Science & Engineering Guangxi Key Laboratory of Information Materials and Guangxi Collaborative Innovation Center of Structure and Property for New Energy and Materials Guilin University of Electronic Technology Guilin 541004 P. R. China

**Keywords:** bifunctional electrocatalysts, bimetallic phosphides, electron redistribution, surface reconstruction, water splitting

## Abstract

Developing high‐efficiency and stable bifunctional electrocatalysts for water splitting remains a great challenge. Herein, NiMoO_4_ nanowires as sacrificial templates to synthesize Mo‐doped NiFe Prussian blue analogs are employed, which can be easily phosphorized to Mo‐doped Fe_2x_Ni_2(1‐x)_P nanotubes (Mo‐FeNiP NTs). This synthesis method enables the controlled etching of NiMoO_4_ nanowires that results in a unique hollow nanotube architecture. As a bifunctional catalyst, the Mo‐FeNiP NTs present lower overpotential and Tafel slope of 151.3 (232.6) mV at 100 mA cm^−2^ and 76.2 (64.7) mV dec^−1^ for HER (OER), respectively. Additionally, it only requires an ultralow cell voltage of 1.47 V to achieve 10 mA cm^−2^ for overall water splitting and can steadily operate for 200 h at 100 mA cm^−2^. First‐principles calculations demonstrate that Mo doping can effectively adjust the electron redistribution of the Ni hollow sites to optimize the hydrogen adsorption‐free energy for HER. Besides, in situ Raman characterization reveals the dissolving of doped Mo can promote a rapid surface reconstruction on Mo‐FeNiP NTs to dynamically stable (Fe)Ni‐oxyhydroxide layers, serving as the actual active species for OER. The work proposes a rational approach addressed by electron manipulation and surface reconstruction of bimetallic phosphides to regulate both the HER and OER activity.

## Graphical abstract

The Mo‐doped Fe_2x_Ni_2(1‐x)_P nanotubes are synthesized and can be directly employed as a superior bifunctional electrocatalyst. The result reveals that the bimetallic FeNiP system with Mo doping can manipulate electron redistribution to optimize the hydrogen adsorption free energy for HER and lead to rapid surface reconstruction to form high active (Fe)Ni‐oxyhydroxide layers for OER, thus endowing extraordinary bifunctional catalytic performance.

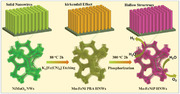

## 1 Introduction

Hydrogen has been considered an important new energy alternative to traditional fossil fuels.^[^ ^1^ ^]^ To date, industrial hydrogen production technologies including coal gasification using fossil fuels, steam reforming, and partial oxidation of heavy hydrocarbons, require large energy consumption, simultaneously along with massive greenhouse gas emissions.^[^ ^2^ ^]^ Therefore, it is desirable to develop an environmentally friendly and low‐cost technology for hydrogen production. At present, alkaline water electrolysis has been regarded as one of the most promising technologies for large‐scale hydrogen production.^[^ ^3^, ^4^, ^5^ ^]^ Commercial Pt/C and IrO_2_‐based materials have been considered to be the most effective cathode and anode catalysts for the electrolysis of water, but their extreme scarcity prevents large‐scale application.^[^ ^6^ ^]^ Hence, non‐noble metal‐based catalytic materials with high efficiency and low cost have aroused great concern for catalyzing hydrogen evolution reaction (HER) and oxygen evolution reaction (OER).

In recent years, the first‐row 3d non‐noble transition metal‐based materials, such as transition metal oxides,^[^ ^7^ ^]^ sulfides,^[^ ^8^, ^9^, ^10^ ^]^ selenides,^[^ ^11^, ^12^, ^13^, ^14^ ^]^ nitrides,^[^ ^15^ ^]^ carbides,^[^ ^16^ ^]^ etc. have been identified as a class of promising catalysts for water splitting. However, most of them cannot function as efficiently bifunctional electrocatalysts for catalyzing both HER and OER, which complicates the construction of electrolyzers and reduces overall electrolytic efficiency. Therefore, more and more research is devoted to exploring highly efficient bifunctional catalysts for both HER and OER in alkaline conditions.^[^ ^17^ ^]^ However, the non‐noble metal‐based catalysts for robust alkaline water electrolysis are still largely limited by the sluggish dynamics of interfacial reactions on the electrodes. For HER, the water dissociation step involved the Volmer step (H_2_O(aq) + e^−^ → H_ad_ + OH^−^) and Heyrovsky step ((H_2_O(aq) + e^−^ + H_ad_ → H_2_ + OH^−^) greatly limits the cathode activity.^[^ ^18^ ^]^ For OER, the sluggish four‐electron transfer accompanied by complex intermediates leads to a very high overpotential at the anode.^[^ ^19^, ^20^, ^21^ ^]^ Thus, how to stimulate the high activities of non‐noble metal‐based bifunctional catalysts by regulating the adsorption‐free energy of intermediates for electrochemical water splitting still faces great challenges.

Recently, transition metal phosphides (TMPs) such as Fe_2_P,^[^ ^22^ ^]^ CoP,^[^ ^23^ ^]^ Ni_2_P,^[^ ^24^ ^]^ etc. based materials, have been regarded as a kind of promising electrocatalysts under alkaline conditions due to their low costs and tunable electronic structures. In general, single‐component Ni_2_P showed high catalytic activity for HER, while CoP or Fe_x_P were found to be effective for OER.^[^ ^25^, ^26^, ^27^ ^]^ Because of this, bimetallic engineering has been proposed to design bifunctional HER/OER catalysts. For example, Han et al.^[^ ^28^ ^]^ reported that bimetallic NiCoP nanocone showed lower overpotentials of 197 and 370 mV at 100 mA cm^−2^ for HER and OER, respectively. It demonstrated that the bimetallic synergies between the different 3d orbitals metal of Ni and Co could regulate the adsorption energies of the intermediates on the surface of the catalysts and enhance the HER/OER performance. In addition, heteroatom doping has been demonstrated as a practical method to manipulate the electron redistribution of TMPs, and thus enhance the catalytic activity.^[^ ^29^ ^]^ For instance, Roh et al.^[^ ^30^ ^]^ synthesized various metals (Fe, Mo, V, Co)‐doped Ni_2_P (NiMP) as overall water‐splitting electrocatalysts. The results indicated that Mo doping can promote the H_2_O dissociation for HER thus giving a lower overpotential, while Fe doping can raise the d‐band center of Ni_2_P thus balancing the adsorption strength of the intermediates in OER. Moreover, previous report has demonstrated that heteroatom doping can efficiently promote the surface reconstruction of electrocatalysts by electron manipulation in the OER.^[^ ^31^ ^]^ Hence, combining bimetallic engineering with heteroatom doping may be a new way to design Ni_2_P‐based catalysts with bifunctional catalytic activities for water electrolysis.

In addition to improving the intrinsic electrocatalytic activities of TMPs by electronic structure modulations, it is more important to design a reasonable nanoarray structure,^[^ ^32^, ^33^, ^34^ ^]^ such as nanowire,^[^ ^35^ ^]^ nanosheet,^[^ ^36^ ^]^ and nanotube,^[^ ^37^ ^]^ which can not only reduce the adhesion of substantial bubbles rapidly generated on the catalysts’ surfaces at large‐current density, but also guarantee the full exposure of active sites and rapidly mass transfer/diffusion of reactants, thus avoiding the activity decrease and shedding of catalysts caused by gas bombardment.^[^ ^6^, ^38^, ^39^ ^]^ Taking into account all these factors offered above, manipulating electron redistribution in Ni_2_P‐based bimetallic nanoarray catalysts through heteroatom doping may be a prospective strategy to stimulate the HER and OER activity. The optimized electron distribution of TMPs could adjust the intermediate‐binding strength for HER, and simultaneously enhance the OER activity by reducing the barrier of reconstruction to accelerate the surface reconstruction to form more active sites, thus thoroughly stimulating the water‐splitting performance.

Herein, the Mo‐doped Fe_2x_Ni_2(1‐x)_P nanotubes (Mo‐FeNiP NTs) on nickel foam were synthesized through an etching process and subsequently, phosphorization method using NiMoO_4_ NWs as sacrificial templates. This synthesis method enables the controlled etching of NiMoO_4_ nanowires that results in a hollow nanotube structure. When utilizing the as‐prepared Mo‐FeNiP NTs as a bifunctional catalyst, it demonstrated superior activity for both HER (*ƞ*_10_  =  30.1 mV, *ƞ*_100 _ =  151.3 mV) and OER (*ƞ*_10_  =  182.5 mV, *ƞ*_100_  =  232.6 mV) in alkaline condition. In addition, the two‐electrode electrolyzer assembled with Mo‐FeNiP NTs as both the anode and cathode electrodes required a very small cell voltage of 1.47 V to reach a current density of 10 mA cm^−2^ and show high stability for 100 h at a relatively high current density of 100 mA cm^−2^. Density functional theory (DFT) calculations and In situ Raman characterization revealed that the bimetallic FeNiP system with Mo doping could manipulate electron redistribution to optimize the hydrogen adsorption free energy for HER and lead to rapid surface reconstruction to form high active (Fe)Ni‐oxyhydroxide layers for OER, thus endowing extraordinary bifunctional catalytic performance. These results suggest that our strategy could provide a promising procedure for designing novel bifunctional electrocatalysts for water splitting with high performance and long durability.

## 2 Results and Discussion

### 2.1 Material Synthesis and Characterization

**Figure 1:**
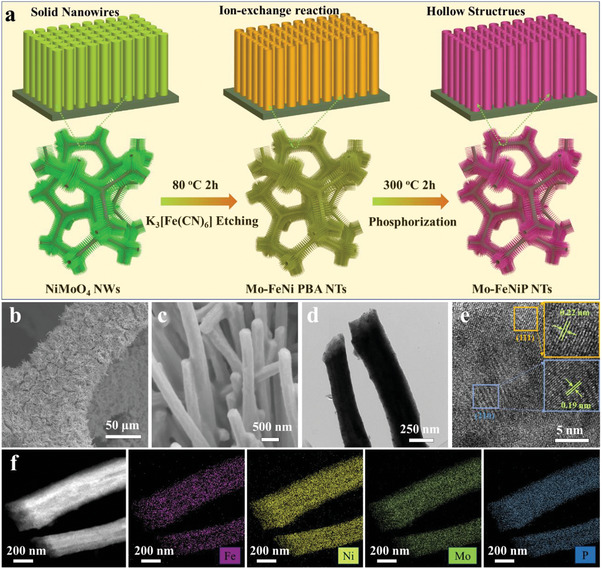
a) The preparation schematic illustration of Mo‐FeNiP NTs/NF. b,c) SEM images of Mo‐FeNiP NTs. d) TEM, and e) HRTEM images of Mo‐FeNiP NTs. f) HAADF‐STEM image and corresponding EDS elemental mappings of Mo‐FeNiP NTs.

As illustrated in **Figure** **1**a, the Mo‐doped bimetallic Fe_2x_Ni_2(1‐x)_P nanotubes array supported on nickel foam (Mo‐FeNiP NTs/NF) were converted from the NiMoO_4_ NWs precursor (NiMoO_4_ NWs/NF) through a controlled ion‐exchange reaction and subsequently phosphorization process. The possible formation and conversion mechanism of porous nanotube structure can be understood as follows (Figure S1, Supporting Information). First, the NiMoO_4_ NWs/NF was fabricated by a hydrothermal method.^[^ ^40^ ^]^ Second, the NiMoO_4_ NWs/NF was immersed in 60 mL of K_3_[Fe(CN)_6_] aqueous solution. During this process, the [Fe(CN)_6_]^3−^ in the solution promoted the etching of the NiMoO_4_ NWs surfaces produced Ni^2+^ and MoO_4_^2−^ ions, and simultaneously accelerated the combing of [Fe(CN)_6_]^3−^ and Ni^2+^ to form more stable K_2_FeNi(CN)_6_ on the surfaces of NiMoO_4_ NWs.^[^ ^41^ ^]^ As the reaction went on, the NiMoO_4_ NWs completely transformed into FeNi‐based Prussian blue analogs (FeNi PBA). Owing to the slow diffusion kinetics, some MoO_4_^2−^ ions remain in the FeNi PBA lattices, thus forming the Mo‐doped FeNi PBA hollow nanotubes (Mo‐FeNi PBA NTs). Third, the Mo‐FeNi PBA NTs were further annealed at 300 °C for 2 h in PH_3_ vapor. In the phosphorization, the Mo‐FeNi PBA NTs reacted with PH_3_ and transformed into Mo‐doped Fe_2x_Ni_2(1‐x)_P nanotubes on NF (Mo‐FeNiP NTs/NF), which can be used as a binder‐free bifunctional catalyst for water splitting.

The microstructures and components of the NiMoO_4_ NWs/NF, Mo‐FeNi PBA NTs/NF, and Mo‐FeNiP NTs/NF were first characterized using electron microscopy. As shown in Figure S2 (Supporting Information) the SEM images reveal the NiMoO_4_ precursor grown on NF shows a uniform solid nanowire shape with a smooth surface. Interestingly, after etching in K_3_[Fe(CN)_6_] aqueous solution, the NiMoO_4_ NWs successfully transformed into Mo‐FeNi PBA NTs, which can be confirmed by SEM and TEM. It can be observed that the orderly nanowire morphology supported on NF was well maintained (Figure S3, Supporting Information). Importantly, the TEM and EDS elemental mapping results show the formation of a hollow interior within the nanowire, which presents a uniform elements distribution of Ni, Fe, Mo, C, and N, indicating the NiMoO_4_ nanowires were in situ transformed into Mo‐FeNi PBA nanotubes (Figure S4, Supporting Information). The HRTEM image shows that the lattice fringes of 0.36 nm correspond to the (220) lattice planes of K_2_FeNi(CN)_6_, which further reveals the formation of Mo‐FeNi PBA NTs (Figure S5, Supporting Information). After phosphorization, it can be seen that both the NiMoP NWs (Figure S6, Supporting Information) and Mo‐FeNiP NTs (Figure 1b,c) catalysts derived from NiMoO_4_ perfectly inherit the nanowire morphology without any noticeable collapse. Furthermore, the TEM image of Mo‐FeNiP NTs (Figure 1d) shows the initial Mo‐FeNi PBA hollow framework remains stable during the phosphorization, which can not only maximize to expose more reactive sites but also efficiently promote the electrolyte access and bubble release for the HER/OER, thus greatly boosting the overall water splitting performance.^[^ ^29^ ^]^ The HRTEM image of Mo‐FeNiP NTs demonstrates that the lattice fringes with the spacing of 0.19 and 0.22 nm correspond to the (210) and (111) crystal planes of standard Ni_2_P.^[^ ^42^, ^43^, ^44^ ^]^ Additionally, the architecture and components of Mo‐FeNiP NTs were further proved by EDS elemental mappings (Figure 1f). It can be seen that the elements of Fe, Ni, Mo, and P are homogeneously distributed on the nanotube structure, confirming the uniform Mo doping in the Fe_2x_Ni_2(1‐x)_P nanotube. Besides, the atomic ratio between the Mo, Fe, and Ni is calculated to be 6:36:58, indicating the low content of Mo in the catalyst. To examine the hollow porous nanotube structure of Mo‐FeNiP NTs/NF, the specific surface area of Mo‐FeNiP NTs/NF and NiMoP NWs/NF were examined by nitrogen adsorption–desorption isotherms (Figure S7, Supporting Information). Compared to NiMoP NWs/NF with a smaller BET surface area of 10.2 m^2^ g^−1^, the Mo‐FeNi PBA NTs/NF presents a larger BET surface area of 24.9 m^2^ g^−1^, which is 2.4 times than that of NiMoP NWs/NF. The high BET surface area could be owing to its hollow porous nanotube structure, which indicates that the porous nanotube structure is indeed formed. Importantly, the porous structure of Mo‐FeNiP NTs/NF can provide more active sites and promote the electrolyte access and bubble release for the HER/OER.^[^ ^12^ ^]^

**Figure 2:**
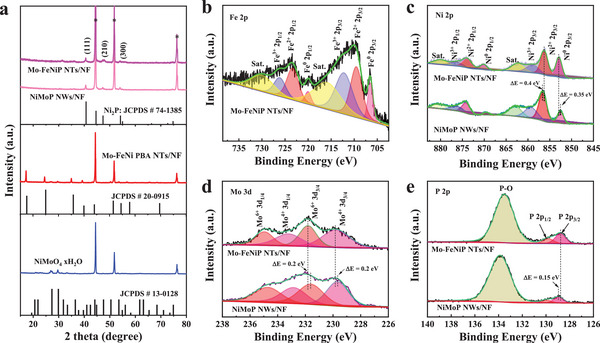
a) XRD patterns of NiMoO_4_ NWs/NF, Mo‐FeNi PBA NTs/NF, NiMoP NWs/NF, and Mo‐FeNiP NTs/NF. The comparison of XPS spectra of NiMoP NWs/NF and Mo‐FeNiP NTs/NF: b) Fe 2p, c) Ni 2p, d) Mo 3d, and e) P 2p.

The crystal structures and chemical states of the samples were analyzed by XRD and XPS techniques. **Figure** **2**a presents the XRD patterns of NiMoO_4_ NWs/NF, Mo‐FeNi PBA NTs/NF, NiMoP NWs/NF, and Mo‐FeNiP NTs/NF. According to a previous report,^[^ ^45^ ^]^ the diffraction peaks of the NiMoO_4_ precursor can be perfectly assigned to the NiMoO_4_·H_2_O (JCPDS No. 13–0128). Notably, the characteristic diffraction peaks of NiMoO_4_ disappeared after the ion‐exchange reaction in the K_2_FeNi(CN)_6_ aqueous solution, and simultaneously some new diffraction peaks appeared, which match well with the standard K_2_FeNi(CN)_6_ (JCPDS No. 20–0915), indicating the NiMoO_4_ precursor completely converted to FeNi PBA. It is noted that NiMoP NWs/NF and Mo‐FeNiP NTs/NF presented similar XRD diffraction peaks. The diffraction peaks with relatively low intensity located at 54.2°, 47.4°, and 40.7° are attributed to the (300), (210), and (111) crystal planes of hexagonal Ni_2_P, respectively (JCPDS No. 74–1385). While the strong peaks appeared at ≈77°, 52°, and 45° originated from the (220), (200), and (111) crystal planes of metallic NF, respectively (JCPDS No. 70–0989, Figure S8, Supporting Information). Compared to the standard Ni_2_P card, the (111) diffraction peak for both the NiMoP NWs and Mo‐FeNiP NTs (Figure S9, Supporting Information) slightly shifts to a lower angle zone, which is due to the lattice expansion caused by the introduction of the larger radius Mo atom.^[^ ^29^ ^]^ Besides, there are no characteristic diffraction peaks for another Mo‐based compound, suggesting the uniform doping of Mo elements in the phosphide samples.^[^ ^27^ ^]^

In addition, the XPS technique was performed to analyze the chemical compositions and the variation of valence states for as‐prepared materials. The XPS surveys (Figure S10, Supporting Information) of Mo‐FeNiP NTs/NF show the characteristic peaks appeared at the binding energies of 134, 233, 284, 531, 712, and 857 eV correspond to the Ni, Fe, Mo, P, C, and O elements, respectively. Notably, an obvious peak of Fe 2p signal located at 857 eV for Mo‐FeNiP NTs/NF appeared, implying the incorporation of Fe element compared to NiMoP NWs/NF. As shown in Figure 2b, the Fe 2p XPS spectrum of Mo‐FeNiP NTs/NF can be deconvoluted into four spin‐orbit couplings. The peaks at binding energies of 709.7, 723.5, 712.3, and 726.2 eV related to Fe^2+^ 2p_3/2_, Fe^2+^ 2p_1/2_, Fe^3+^ 2p_3/2_, and Fe^3+^ 2p_1/2_,^[^ ^46^ ^]^ respectively. While the peaks appeared at 706.7 (Fe^0^ 2p_3/2_) and 720.1 (Fe^0^ 2p_1/2_) eV represented the metallic Fe─P bonding,^[^ ^26^ ^]^ demonstrating the existence of Fe─P configuration in Mo‐FeNiP NTs. Moreover, the two accompanied peaks at 716.0 and 730.1 eV corresponded to the satellite peak of Fe 2p. In the Ni 2p spectrum, the two peaks located at 852.9 and 870.2 eV are assigned to the Ni^0^ 2p_3/2_ and Ni^0^ 2p_1/2_ (Figure 2c), respectively, which relate to the electronic state of metallic Ni─P bonding in Mo‐FeNiP NTs.^[^ ^47^ ^]^ The two spin‐orbit couplings of peaks appearing at 856.3 (or 858.9) eV and 873.9 (or 876.6) eV are ascribed to the Ni^2+/3+^ oxidized species. In addition, the peaks at 862.5 and 880.2 eV correspond to the shakeup satellite peaks of Ni 2p.^[^ ^37^ ^]^ It's worth noting that the position of Ni^0^ peaks shifted to higher binding energy, whereas the position of Ni^2+^ peaks shifted to lower binding energy for Mo‐FeNiP NTs compared to NiMoP NWs, indicating strong electron interactions.^[^ ^48^ ^]^ This result may be caused by the Mo doping which could tune the electronic structure of Fe_2x_Ni_2(1‐x)_P. Besides, similar to NiMoO_4_ NWs, the high‐resolution Mo 3d spectrum reveals the presence of residual Mo species (+6) in Mo‐FeNi PBA NTs (Figure S11, Supporting Information). In Figure 2d, the Mo 3d spectrum of Mo‐FeNiP NTs can be divided into two couple of spin‐orbit features. The peak couple with larger peak area observed at 229.8 and 233.3 eV are ascribed to Mo^4+^ 3d_3/4_ and Mo^4+^ 3d_3/4_,^[^ ^49^ ^]^ whereas the two peaks appearing at 231.8 and 234.9 eV correspond to Mo^6+^ 3d_3/4_ and Mo^6+^ 3d_3/4_,^[^ ^40^ ^]^ respectively. Compared to Mo‐FeNi PBA NTs, the main valence state of Mo in Mo‐FeNiP NTs transformed to the + 4 states, which is associated with the formation of the Mo bond to P.^[^ ^29^ ^]^ Furthermore, Figure 2e illustrates the P 2p spectra exhibited a spin‐orbit coupling of peaks at binding energy of 128.8 (P 2p_3/2_) and 129.7 (P 2p_1/2_) eV, attributing to the metal─P bonding, whereas a broad peak observed at 133.5 eV is ascribed to the P─O bonding, relating to the surface oxide layers of metal phosphides.^[^ ^26^ ^]^ As compared with NiMoP NWs, the P 2p shifted to a lower binding energy, while Mo 3d shifted to a higher binding energy, suggesting that the electrons may transfer from Mo and Ni to Fe and P sites. The XPS results indicated that the formation of Mo‐doped Fe_2x_Ni_2(1‐x)_P nanotube structure with strongly electronic tuning could be beneficial to promote the OER and HER performance.^[^ ^30^ ^]^

### 2.2 Electrochemical Measurement of OER and HER Activities

**Figure 3:**
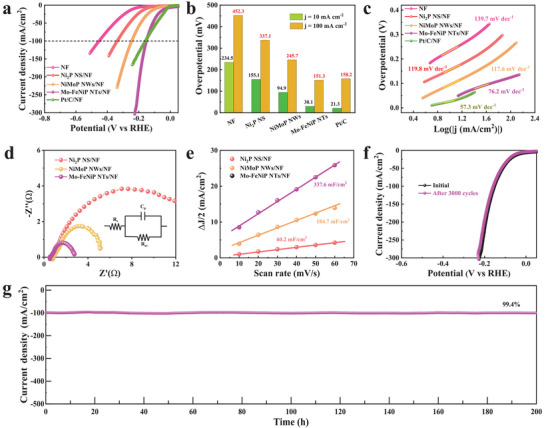
The HER performance of Mo‐FeNiP NTs/NF compared with NF, Ni_2_P NS/NF, NiMoP NWs/NF, and RuO_2_/NF electrocatalysts: a) the LSV curves, b) the overpotential comparisons at current densities of 10 and 100 mA cm^−2^, and c) Tafel slopes. d) EIS Nyquist plots, and e) Estimated *C*_dl_ of Ni_2_P NS/NF, NiMoP NWs/NF, and Mo‐FeNiP NTs/NF. f) the LSV curves before and after HER catalysis. g) Chronoamperometric current density curve of Mo‐FeNiP NTs/NF at a current density of 100 mA cm^−2^.

To examine the water‐splitting performance of as‐synthesized electrocatalysts, the HER/OER activities were first evaluated in 1 m KOH electrolyte. As displayed in **Figure** **3**a, the HER performance of Mo‐FeNiP NTs/NF, NiMoP NWs/NF, Ni_2_P NS/NF, and Pt/C/NF electrodes was investigated by using linear sweep voltammetry (LSV). In all the samples, the Mo‐FeNiP NTs/NF show a robust HER activity compared to the control samples of Ni_2_P NS/NF and NiMoP NWs/NF. It showed a dramatically lower overpotential of 30.1 mV at 10 mA cm^−2^ (𝜂_10_), which is close to commercial Pt/C/NF (21.3 mV), and lower than Ni_2_P NS/NF (155.1 mV) and NiMoP NWs/NF (94.9 mV), respectively (Figure 3b). These exceptional HER activities of Mo‐FeNiP NTs/NF are obtained due to the formation of the Fe_2x_Ni_2(1‐x)_P hollow nanostructure with Mo doping, which can not only tune the electron structure but also boost the exposure of active sites. Furthermore, a small overpotential of 151.3 mV is required for Mo‐FeNiP NTs/NF even at 100 mA cm^−2^ (Figure 3b), which is lower than that of commercial Pt/C/NF (158.2 mV), and far better than that of Ni_2_P NS/NF (337.1 mV) and NiMoP NWs/NF (245.7 mV). Based on these results, structural optimization coupled with electronic tuning significantly enhances the transport of ions/electrons for the HER reaction. The Tafel slopes as a useful tool were extracted from corresponding LSV curves to uncover the rate‐limitation mechanism of catalysts.^[^ ^50^ ^]^ Figure 3c shows that the Tafel slope of Mo‐FeNiP NTs/NF was 76.2 mV dec^−1^, which is lower than that of 119.8 mV dec^−1^ (Ni_2_P NS/NF) and 117.6 mV dec^−1^ (NiMoP NWs/NF), and comparable to that of commercial Pt/C/NF (57.3 mV dec^−1^). These demonstrated that Mo‐FeNiP NTs/NF as an efficient electrocatalyst follows the Volmer reaction mechanism for HER. Additionally, an electrochemical impedance spectroscopy (EIS) technique was employed to examine the charge transfer resistance of different samples at the catalyst/electrolyte interface. As shown in Figure 3d, the fitted *R*_ct_ value from Nyquist plots for Mo‐FeNiP NTs/NF is only 2.1 Ohms, which is lower than those of NiMoP NWs/NF (4.7 Ohms) and Ni_2_P NS/NF (14.5 Ohms), demonstrating the preferable HER kinetics of the Mo‐FeNiP NTs. To assess the electrochemically active surface area (ECSA), the electrochemical double‐layer capacitances (*C*_dl_) were calculated from the corresponding cyclic voltammograms (Figure S12, Supporting Information). As shown in Figure 3e, the Mo‐FeNiP NTs/NF present the largest *C*_dl_ value of 337.6 mF cm^−2^ in comparison to Ni_2_P NS/NF (60.2 mF cm^−2^) and NiMoP NWs/NF (184.7 mF cm^−2^), indicating the hollow nanotube structure can expose more active sites for HER. These results suggest the positive synergy effect between the electron redistribution tuned by Mo doping and hollow nanostructure increased exposed catalytic active sites for hydrogen adsorption. In particular, the high‐performance Mo‐FeNiP NTs/NF is one of the best transition metals phosphide catalysts for HER ever reported, which surpasses most state‐of‐the‐art electrocatalysts in alkaline media (Table S1, Supporting Information). In order to use the synthesized catalysts for industrial applications, the long‐term stability of Mo‐FeNiP NTs/NF was examined by using a chronoamperometry method at a fixed overpotential of 155 mV (Figure 3g). The Mo‐FeNiP NTs/NF demonstrated remarkable HER stability at a large current density of 100 mA cm^−2^ with a high current retention of 99.4% after 200 h electrolysis, demonstrating the industrial application potential of the catalyst. Besides, the polarization curve measured after 3000 CV sweeps shows a marginal decrease compared to the initial curve (Figure 3f), indicating minor corrosion of the catalyst during the HER process. To further investigate the compositional evolution following the HER test, the characterization analysis of XPS was performed. (Figure S13, Supporting Information). It is noted that the binding energies of Fe 2p, Ni 2p, Mo 3d, and P 2p did not change significantly after the HER catalysis, revealing that Mo‐FeNiP NTs did not undergo surface changes during the HER process. Based on the observations, we determined that Mo‐FeNiP NTs act as the actual active sites for HER.

**Figure 4:**
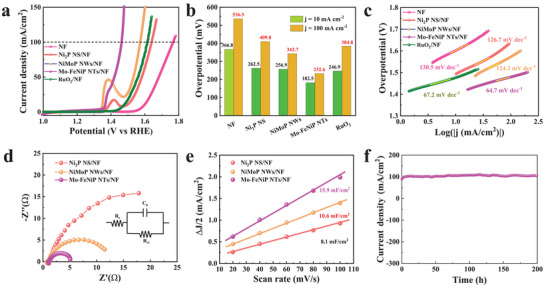
The OER performance of Mo‐FeNiP NTs/NF compared with NF, Ni_2_P NS/NF, NiMoP NWs/NF, and RuO_2_/NF electrocatalysts: a) the LSV curves, b) the overpotential comparisons at current densities of 10 and 100 mA cm^−2^, and c) Tafel slopes. d) EIS Nyquist plots, and e) Estimated C_dl_ of Ni_2_P NS/NF, NiMoP NWs/NF, and Mo‐FeNiP NTs/NF. f) Chronoamperometric current density curve of Mo‐FeNiP NTs/NF at current density of 100 mA cm^−2^.

In addition to the HER, the OER performances of the catalysts were also examined by the LSV in 1 m KOH. As displayed in **Figure** **4**a, the current density of Mo‐FeNiP NTs/NF in LSV rises faster compared to the Ni_2_P NS/NF, NiMoP NWs/NF, NF substrate, and commercial RuO_2_/NF. The overpotentials of Mo‐FeNiP NTs/NF at current densities of 10 and 100 mA cm^−2^ are 182.5 and 232.6 mV (Figure 4b; Figure S14, Supporting Information), respectively, which are much smaller than those of Ni_2_P NS/NF (262.5 and 409.8 mV), NiMoP NWs/NF (256.9 and 342.7 mV), NF substrate (366.8 and 536.5 mV), and commercial RuO_2_/NF (246.9 and 384.8 mV). Correspondingly, the Mo‐FeNiP NTs/NF shows the smallest Tafel slope of 64.7 mV dec^−1^ compared to those of 126.7, 124.3, and 67.2 mV dec^−1^ for Ni_2_P NS/N, NiMoP NWs/NF, and RuO_2_/NF (Figure 4c), respectively, indicating its highly intrinsic OER kinetics. Similar to HER, the Mo‐FeNiP NTs/NF shows the smallest *R*_ct_ value of only 3.9 Ohms (Figure 4d), which is much lower than that of 12.1 Ohms for Ni_2_P NS/NF and 45.2 Ohms for NiMoP NWs/NF. Furthermore, it also presents the largest *C*_dl_ value of 15.9 mF cm^−2^ (Figure 4d; Figure S15, Supporting Information) compared to Ni_2_P NS/NF (8.1 mF cm^−2^) and NiMoP NWs/NF (10.6 mF cm^−2^). These results indicate that the hollow nanotube structure with Mo doping is the most effective way to develop highly efficient OER catalysts, which can not only promote electron transfers in the OER process but also provide a large number of active sites.^[^ ^30^ ^]^ In addition, we suspect that the Mo doping may promote a rapid surface reconstruction to further boost the OER performance. Surprisingly, the excellent OER performance of Mo‐FeNiP NTs/NF surpasses most of the FeNi‐based catalysts reported so far (Table S2, Supporting Information). Furthermore, the chronoamperometry test shows there is no significant deterioration that can be noticed after 200 h operating (Figure 4f). The LSV curves (Figure S16, Supporting Information) of Mo‐FeNiP NTs/NF before and after the OER catalysis for 3000 cycles show negligible overpotential loss, further demonstrating excellent durability under alkaline OER.

### 2.3 Mechanism for the Enhancement of HER and OER

**Figure 5:**
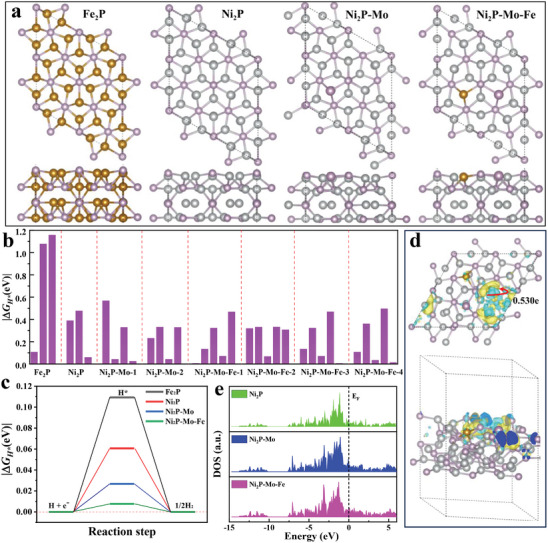
a) The optimal models of Fe_2_P, Ni_2_P, Ni_2_P‐Mo, and Ni_2_P‐Mo‐Fe (Top view and side view). b) The absolute value of adsorption Gibbs free energy change on all possible active sites on various models. c) The three‐state free energy diagram on the best active sites for the optimal models. d) Differential charge distribution and Bader charge transfer of the optimal dual‐doped Ni_2_P‐Mo‐Fe model on the best active site. Blue color indicates positive charge and yellow color indicates negative values of electron quantities. The isosurface value is set to 0.004 e Bohr^−3^. The red arrow indicates the direction of charge transfer (Top view and side view). e) The changing trend of DOSs for the optimal structural models.

To investigate insights into the enhanced mechanism that underlies the high activities of Mo‐FeNiP NTs for HER, systematic DFT calculations were performed to uncover the effects of Mo and Fe in the catalysis. As shown in **Figure** **5**a, four different calculation models including Fe_2_P, Ni_2_P, Mo‐doped Ni_2_P (Ni_2_P‐Mo), and Fe and Mo co‐doped Ni_2_P (Ni_2_P‐Mo‐Fe) were established to cover virtually all experimentally available cases. All 34 possible active sites for HER on the proposed eight models (Figures S17–S20 and Table S4, Supporting Information) were surveyed to find the ‘best’ sites for HER for each model, as shown in Figure 5b. The optimized H^*^ adsorption models of Fe_2_P, Ni_2_P, Ni_2_P‐Mo, and Ni_2_P‐Mo‐Fe (Figures S17b, S18d, S19e, and S20b, Supporting Information) presented that H^*^ is adsorbed on P sites of Fe_2_P and Ni hollow sites of Ni_2_P, Ni_2_P‐Mo, and Ni_2_P‐Mo‐Fe, respectively. Furthermore, the hydrogen adsorption strength on these optimal sites was investigated to depict the Gibbs free energy (*ΔG*_H∗_) diagrams (Figure 5c). Compared to Fe_2_P, Ni_2_P, and Ni_2_P‐Mo models, the lowest value of |*ΔG*_H∗_| was obtained on the Ni_2_P‐Mo‐Fe model, implying that the introduction of Mo and Fe elements could synergistically promote hydrogen adsorption,^[^ ^32^ ^]^ which was in line with experimental results. To further explore the correlation between catalytic property and electronic structure, Bader charge transfers and three density of states (DOS) were computed on each Ni_2_P‐based optimal model.^[^ ^51^ ^]^ Figure S21 (Supporting Information) and Figure 5d show the difference in charge density on the Ni hollow site of Ni_2_P, Ni_2_P‐Mo, and Ni_2_P‐Mo‐Fe models, respectively. All the atoms in the Ni_2_P model are in a relative equilibrium state. Heterogeneous atoms and chemisorption site on nickel phosphide with their respective electronic states can spilt into bond orbital and anti‐bond orbital through interaction, and at this point, metal‐phosphorus 3d‐2p hybrid state with new electronic state compared with prior nickel phosphide without heterogeneous atoms with group molecule at the chemical reaction to form new bond orbital σ and anti‐bond orbital σ^*^. The adsorption strength of the group molecule on the active site is governed by the filling degree of new anti‐bond orbital σ^*^. Only when the new anti‐bond orbital has an appropriate filling degree of electrons, can the adsorption strength reach the optimal level of neither strong nor weak.^[^ ^51^ ^]^ Therefore, when introducing the hetero‐atoms into the Ni_2_P model, electrons tend to be redistributed around the P atom, thus reaching a new equilibrium.^[^ ^52^ ^]^ Especially, whether introducing the single‐doped Mo or dual‐doped Fe and Mo atoms into Ni_2_P, the charge density around P increases, while the charge density around Ni decreases due to strong electron redistribution. compared to Ni_2_P‐Mo (0.543e), a negative charge transfer was detected at the hollow Ni site of Ni_2_P‐Mo‐Fe (0.530e), which is inconsistent with the XPS results. This electron manipulation of Ni_2_P results in enhanced electron interaction, which in turn adjusts the *ΔG*_H∗_ of the hollow Ni site, therefore improving electrochemical HER performance.^[^ ^27^ ^]^ In addition, the Ni_2_P‐Mo‐Fe near the Fermi level shows a stronger DOS in comparison to that of Ni_2_P and Ni_2_P‐Mo (Figure 5e), indicating that the introduction of Fe in Ni_2_P‐Mo‐Fe could enhance the intrinsic electrical behaviors of the catalyst.^[^ ^53^ ^]^

**Figure 6:**
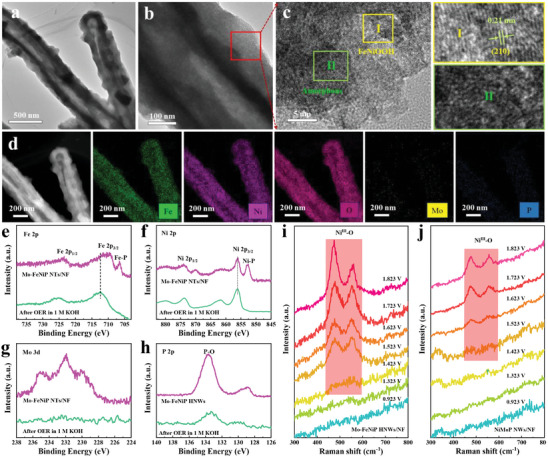
a,b) TEM images, c) HRTEM image, and d) HAADF‐STEM image with corresponding EDS elemental mappings of Mo‐FeNiP NTs after 3000 CV cycles. The XPS spectra of Mo‐FeNiP NTs measured before and after 3000 CV cycles: e) Fe 2p, f) Ni 2p, g) Mo 3d, and h) P 2p. In situ Raman spectra of i) Mo‐FeNiP NTs, and j) NiMoP NWs with the anodic potential ranging from 0.923 to 1.823 V (vs RHE).

As for OER, as we all know most transition metal‐based electrocatalysts undergo irreversible surface phase transition during OER.^[^ ^54^ ^]^ Thus, we examined the microstructures, surface compositions, and chemical states to determine the real active sites for the high OER activities of Mo‐FeNiP NTs, after the OER catalysis for 3000 cycles in 1.0 m KOH by TEM, XPS, Raman, and EDX experiments. As shown in **Figure** **6**a, the hollow nanotube structure of Mo‐FeNiP NTs is perfectly maintained after the OER catalysis. The enlarged image of the nanotube (Figure 6b) exhibits an amorphous layer formed on the nanotube surface. Furthermore, the HRTEM image of Mo‐FeNiP NTs after OER testing reveals the presence of an amorphous nanocrystalline layer on the nanotube surface (Figure 6c). The lattice fringes of 0.21 nm could be assigned to the (210) crystal plane of Ni oxyhydroxides (NiOOH). The EDS elemental mappings after OER testing (Figure 6d) show the main components of the whole nanotube include the Ni, Fe, and O elements, while the percentage composition of Mo and P elements is almost near zero, which suggests a deep reconstruction occurred during the OER oxidation process.^[^ ^55^ ^]^ In addition, the XPS spectra of Mo‐FeNiP NTs after OER testing further demonstrate the surface transformation of Mo‐FeNiP to (Fe)NiOOH. The Fe 2p (Figure 6e) and Ni 2p (Figure 6f) peaks in Mo‐FeNiP NTs show a positive shift after OER testing, indicating they are oxidized to higher valence states. Besides, the signals of Mo 3d (Figure 6g) and P 2p (Figure 6h) spectra almost disappeared after the OER test, which suggests the dissolving of Mo atoms in the electrolyte during the reconstruction process. It shows that the incorporation of Fe and the dissolving of Mo can synergistically promote the surface reconstruction of Mo‐FeNiP NTs, thus enhancing the OER catalytic activity. The in situ Raman spectra of Mo‐FeNiP NTs and NiMoP NWs catalysts were further performed on an applied voltage ranging from 0.923 to 1.823 V (vs RHE) to elucidate their dynamic reconstruction behaviors during the OER catalysis. It can be seen that the Raman spectrum of the Mo‐FeNiP NTs shows no obvious surface change <1.323 V vs. RHE (Figure 6i). When the applied potential increases to 1.423 V vs. RHE, there are two characteristic Raman peaks were detected at around 477 and 559 cm^−1^, which are assigned to the *E*_g_ and *A*_1g_ vibration modes of Ni^III^─O in NiOOH,^[^ ^56^ ^]^ respectively, indicating the beginning of the surface reconstruction of Mo‐FeNiP NTs. It is worth noting that the Raman intensities of the *E*_g_ and *A*_1g_ peaks become much higher with the increasing of potential from 1.423 to 1.823 V, indicating the deep reconstruction in the OER process. Notably, there are no Raman characteristic peaks of Fe^III^─O vibrations appearing in Mo‐FeNiP NTs, thus we conclude that the Fe atoms are uniformly doped into NiOOH. Moreover, the reconstruction potential of Mo‐FeNiP NTs is 1.423 V vs. RHE is significantly lower than that of 1.623 V vs. RHE for NiMoP NWs (Figure 6j), implying the dissolving of Mo boosts the rapid surface reconstruction of Mo‐FeNiP NTs. Thus, the above TEM, XPS, and in situ Raman analysis mutually revealed the rapid surface reconstruction of Mo‐doped Fe_2x_Ni_2(1‐x)_P NTs to dynamically stable (Fe)Ni‐oxyhydroxide layers ((Fe)NiOOH), which act as the actual active species to participate the OER process.

### 2.4 Electrocatalytic Performance for Overall Water Splitting

**Figure 7:**
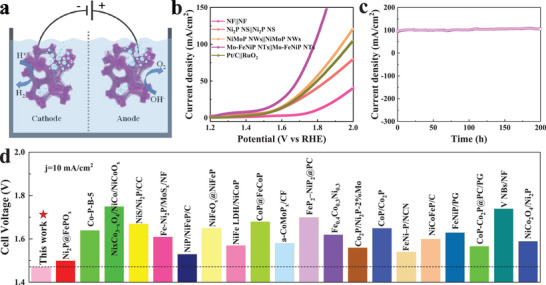
a) Schematic illustration of the two‐electrode cell of Mo‐FeNiP NTs(+)||Mo‐FeNiP NTs(‐). b) Polarization plots of Mo‐FeNiP NTs(+)||Mo‐FeNiP NTs(‐) and other contrast electrolyzers. c) Durability test of Mo‐FeNiP NTs(+)||Mo‐FeNiP NTs(‐) electrolyzer at a current density of 100 mA cm^−2^. d) Comparison of Mo‐FeNiP NTs(+)||Mo‐FeNiP NTs(‐) electrolyzer with the reported cell voltages at 10 mA cm^−2^. The symbols of ‘‘+” and ‘‘‐’’ represent the anode and cathode, respectively.

Encouraged by high activity and stability for both the HER and OER, the Mo‐FeNiP NTs/NF are hypothesized to be used as a promising electrocatalyst for overall water splitting. Thus, we constructed a two‐electrode electrolyzer using Mo‐FeNiP NTs as both anode and cathode for investigating the performance of water splitting in the alkaline electrolyte (**Figure** **7**a). For comparison, the NF(+)||NF(‐), NiMoP NWs(+)||NiMoP NWs(‐), Ni_2_P NS(+)||Ni_2_P NS(‐), and RuO_2_(+)||Pt/C(‐) electrolyzers were also assembled and tested at the same condition. As shown in Figure 7b, the NF(+)||NF(‐) electrolyzer shows a high cell voltage of 1.8 V at 10 mA cm^−2^, indicating inferior electrocatalytic performance. Notably, the Mo‐FeNiP NTs(+)||Mo‐FeNiP NTs(‐) electrolyzer only needs a lower cell voltage of 1.47 V to afford 10 mA cm^−2^, which is considerably smaller than those of NiMoP NWs (1.55 V), Ni_2_P NS (1.64 V) and RuO_2_||Pt/C (1.57 V) based electrolyzers. In particular, Mo‐FeNiP NTs‐based electrolyzer outperforms most previously reported non‐noble electrocatalysts for overall water splitting (Figure 7d; Table S3, Supporting Information), such as NiCoP@NC NA/NF (156 V),^[^ ^57^ ^]^ Fe_0.4_Co_0.3_Ni_0.3_ (1.62 V),^[^ ^2^ ^]^ a‐CoMoP_x_/CF (1.58 V),^[^ ^58^ ^]^ and so on. In addition, the stability of Mo‐FeNiP NTs(+)||Mo‐FeNiP NTs(‐) electrolyzer for water electrolysis was performed in 1 m KOH at a relatively large current density of 100 mA cm^−2^. As we can see from Figure 7c, this electrolyzer almost remains stable for 200 h during water electrolysis. These electrolysis cell evaluation results demonstrate the superiority and feasibility of Mo‐FeNiP NTs electrocatalyst for water splitting. Thus, we concluded that our Mo‐FeNiP NTs catalyst can be employed as a promising bifunctional electrocatalyst for alkaline water electrolysis.

## 3 Conclusion

In summary, a well‐defined Mo‐doped bimetallic Fe_2x_Ni_2(1‐x)_P nanotubes (Mo‐FeNiP NTs) on nickel foam has been achieved through an etching process and subsequently phosphorization method by using NiMoO_4_ nanowire as a template. The Mo‐FeNiP NTs exhibit excellent electrocatalytic activities toward HER (*η*_100_ = 151.3 mV) and OER (*η*_100_ = 232.6 mV). Importantly, the two‐electrode electrolyzer using Mo‐FeNiP NTs as both the anode and cathode catalyst only needs a small cell voltage of 1.47 V to attain 10 mA cm^−2^ and shows long‐term operation stability of 200 h. The characterization results revealed that the bimetallic hollow nanotubes with Mo doping synergistically endow Mo‐FeNiP NTs with abundant exposed active sites, faster mass diffusions/bubble releases, and adjustable electronic structures for overall water catalysis. DFT calculations and in situ Raman characterization further confirmed that the Mo doping can efficiently manipulate the electron redistribution for HER, and simultaneously promote a rapid surface reconstruction caused by the fast dissolving of Mo during the OER process, which could optimize the adsorption free energy of intermediates on the real active sites for HER and OER, resulting in extraordinary bifunctional catalytic performance. This work not only contributes to the development of high‐efficiency bifunctional electrocatalysts for water splitting but also deep insight into the exploration of activity enhancement of transition metal‐based phosphide electrocatalysts.

## 4 Experimental Section

The experimental details are reported in the Supporting Information.

## Conflict of Interest

The authors declare no conflict of interest.

## Supporting information

Supporting Information

## Data Availability

Research data are not shared.
