# Supplementary material for: Electron Manipulation and Surface Reconstruction of Bimetallic Iron–Nickel Phosphide Nanotubes for Enhanced Alkaline Water Electrolysis
Source: Adv Sci (Weinh). 2024 May 5;11(26):2401207. doi: 10.1002/advs.202401207 (PMC11234420; doi:10.1002/advs.202401207)
Supplement: Supplementary file 1 — Supporting Information [file ADVS-11-2401207-s001.pdf]

ADVANCED SCIENCE
Open Access

Supporting Information

for *Adv. Sci.*, DOI 10.1002/advs.202401207

Electron Manipulation and Surface Reconstruction of Bimetallic Iron–Nickel Phosphide Nanotubes for Enhanced Alkaline Water Electrolysis

Xinqiang Wang, Jinhao Zhou, Wengang Cui, Fan Gao, Yong Gao, Fulai Qi, Yanxia Liu, Xiaoying Yang, Ke Wang, Zhenglong Li, Yaxiong Yang, Jian Chen, Wenping Sun, Lixian Sun and Hongge Pan**

Supporting Information

Electron Manipulation and Surface Reconstruction of Bimetallic Iron–Nickel Phosphide Nanotubes for Enhanced Alkaline Water Electrolysis

Xinqiang Wang, Jinhao Zhou, Wengang Cui, Fan Gao, Yong Gao, Fulai Qi, Yanxia Liu, Xiaoying Yang, Ke Wang, Zhenglong Li, Yaxiong Yang, Jian Chen, Wenping Sun, Lixian Sun and Hongge Pan**

X. Q. Wang, W. G. Cui, F. Gao, Y. Gao, F. L. Qi, Y. X. Liu, X. Y. Yang, K. Wang, Z. L. Li, Y. X. Yang, J. Chen, Prof. H. G. Pan
Institute of Science and Technology for New Energy Xi'an Technological University, Xi'an 710021, China
E-mail: honggepan@zju.edu.cn, hgpan@zju.edu.cn

J. H. Zhou
Guangdong-Hong Kong-Macao Joint Laboratory for Intelligent Micro-Nano Optoelectronic Technology, School of Physics and Optoelectronic Engineering, Foshan University, Foshan, 528225, P. R. China

W. P. Sun
School of Materials Science and Engineering State Key Laboratory of Clean Energy Utilization, Zhejiang University, Hangzhou, 310027, P. R. China

L. X. Sun
School of Material Science & Engineering, Guangxi Key Laboratory of Information Materials and Guangxi Collaborative Innovation Center of Structure and Property for New Energy and Materials, Guilin University of Electronic Technology, Guilin, 541004, P. R. China

This file includes:

1. **Experimental section 1-8**
2. **Supplementary Figures S1-21**
3. **Supplementary Tables S1-4**
4. **Supplementary References**

1. Experimental section

1.1. Synthesis of $NiMoO_4$ NWs/NF

The $NiMoO_4$ nanowires grown on nickel foam ($NiMoO_4$ NWs/NF) were fabricated by a hydrothermal method. 2.5 mmol of $NaMoO_4 \cdot 2H_2O$ and $NiCl_2$ $6H_2O$ were dissolved in 60mL DI water. After vigorous stirring for 20 min, a piece of cleaned nickel foam (2 cm × 5 cm) was immersed into the obtained solution and then maintained at 150°C for 6 h. After cooling down to room temperature, the $NiMoO_4$ NWs/NF was washed with DI water and ethanol several times, followed by drying at 60°C for 12 h.

1.2. Synthesis of Mo-FeNi PBA NTs/NF

The as-synthesized $NiMoO_4$ NWs/NF (2 cm × 5 cm) was put in a 100 mL Teflon-lined stainless-steel autoclave containing 100 mg $K_3[Fe(CN)_6]$ aqueous solution (60 mL), and the second hydrothermal reaction was carried out at 80 °C for 2 h. Subsequently, the sample was carefully rinsed with DI water and ethanol at least 5 times and dried at 60 °C for 12 h to obtain Mo doped FeNi based Prussian blue analogs nanotubes on nickel foam (Mo-FeNi PBA NTs/NF).

1.3. Synthesis of Mo-FeNiP NTs/NF

The Mo-FeNiP NTs/NF were synthesized through a typical phosphating process. 1g $NaH_2PO_2\cdot$powders and a piece of Mo-FeNi PBA NTs/NF (2 cm × 5 cm) were placed at the upstream and downstream side of a two-temperature zone tube furnace, respectively. Afterward, the upstream and downstream zones were heated to 280 °C and 350 °C within 140 min under a high-purity Ar atmosphere and then maintained for 2 h. After cooling down to room temperature, the Mo-FeNiP NTs/NF was obtained.

1.4. Synthesis of Ni_2P NS/NF

Typically, 1.4 g of hexamethylenetetramine and 1.45 g of nickel nitrate hexahydrate ($Ni(NO_3)_2 \cdot 6H_2O$) were dissolved in 60 mL of deionized water at ambient temperature by vigorous stirring for 15 minutes. Then, a piece of cleaned NF (2 cm × 5 cm) was subsequently

immersed into the as-obtained solution and maintained at 100 °C for 10 h. After cooling to room temperature, the $Ni(OH)_2$ nanosheets on nickel foam ($Ni(OH)_2$ NS/NF) were obtained by sonicating and washing in deionized water and ethanol, respectively. Finally, the as-papered $Ni(OH)_2$ NS/NF was annealed at 350 ^oC for 2 h in a tube furnace, together with the solid sodium hypophosphite (1 g) at the upstream side under the Ar atmosphere.

1.5. Synthesis of Pt/C or RuO_2/NF

To prepare the Pt/C or RuO_2 on NF (RuO_2/NF or Pt/C/NF) electrodes, 50 mg of the commercial Pt/C (20 wt%) and RuO_2 powders were dispersed in 950 μL of mixture solution of water/ethanol (v/v, 1:1). After sonication for 30 minutes, 50 μL of Nafion solution (5 wt %) was added into the mixture solution. Then, a homogeneous ink was obtained by continuous sonication for at least 30 minutes. Finally, a certain amount of the catalyst ink was dropped onto nickel foam with a working area of 1×1 cm^2, which can be directly served as the working electrodes.

1.6. Characterizations

The crystalline structures of $NiMoO_4$ NWs/NF, Mo-FeNiP NTs/NF, etc., were examined by X-ray diffraction (XRD, Rigaku diffractometer). The chemical states of Mo-FeNiP NTs/NF and other control samples were investigated by using an X-ray photoelectron spectroscopy (XPS, Al Kα radiation). Scanning electron microscopy (SEM) was used to examine the microstructures of as-prepared samples (Tescan, Amber). Besides, transmission electron microscopy (TEM) measurements were carried out on a Talos F200S analyzer at 200 kV to further examine the microstructure and crystal structure of as-prepared samples.

1.7. Measurements of Electrocatalytic Properties

A CHI670D electrochemical workstation was used to investigate the electrocatalytic properties of the samples in N_2-saturated 1.0 M KOH electrolyte. A graphite rod and a standard Hg/HgO electrode were used as the counter and reference electrode, respectively. Meanwhile, the NF substrate, Ni_2P NS/NF, NiMoP NWs/NF, Mo-FeNiP NTs/NF, Pt/C/NF, or RuO_2/NF with an area of 1 cm^2 was employed as the working electrode. The line scan voltammograms (LSV) curves for HER were recorded with a scanning rate of 5 mV s^{-1}, while the LSV curves for OER and overall water splitting were recorded at a relatively slow scanning rate of 2 mV s^{-1}. The voltammograms for HER or OER were recorded at the voltage range of 0.05 to 0.25 V or 0.9 to 1 V (vs RHE) by using cyclic voltammetry. The electrochemical impedance spectroscopy (EIS) for HER and OER was measured at overpotentials of 80 and 200 mV (vs RHE) in the frequency range of 0.1 Hz-100 kHz, respectively. The durability of Mo-FeNiP NTs/NF was estimated by chronoamperometry at

different voltages for HER, OER, and overall water splitting. In this work, all the potential values were iR-compensated and calibrated to reversible hydrogen electrodes (RHE): $E_{RHE} = E_{Hg/HgO} + 0.923 - iR$.

1.8. Computational details

1.8.1. The change of adsorption energy and Gibbs free energy of H chemisorption on active sites

Despite two mechanisms including Volmer–Heyrovsky and Volmer–Tafel for HER, the H adsorption step is the key one for both V-T and V-H mechanisms. Consequently, this first step was studied to estimate the properties of catalyst. In the process of HER, H^+ ions are adsorbed on active sites of catalyst surface under external electric potential U. Therefore, the process can be written as

$$H^{+} + \boldsymbol{e}^{-} + * \rightarrow H^{*} \quad \text{(S1)}$$

where * refers to adsorption site on catalyst surface, and H and H^* represent the free H^+ ions and H^+ adsorbed to sites, respectively. Therefore, the hydrogen (H) adsorption on various catalyst surface is able to be defined as

$$\Delta E = E(H + *) - E(H) - E(*)$$
(S2)

where $E(H + *)$ and $E(*)$ are the energy of catalyst with/without H chemisorption, respectively, and $E(H)$ is the energy of H and can be given as $E(H) = 1/2E(H_2)$.

The Gibbs free energy for hydrogen adsorption (ΔG_{H*}) is able to be calculated by taking zero point energy and entropy corrections into account such that

$$\Delta G_{H*} = \Delta E + \Delta E_{ZPE} - T\Delta S$$
(S3)

where ΔE_{ZPE} and ΔS are the difference in zero point energy and entropy between the adsorbed hydrogen and hydrogen in the gas phase, respectively. As the vibrational entropy of H^* in the adsorbed state is small, the entropy of adsorption of $1/2H_2$ is $\Delta S_H \approx -1/2S^0_{H_2}$, where $S^0_{H_2}$ is the entropy of H_2 in the gas phase at standard conditions. It is concluded that the overall corrections are

$$\Delta G_{H*} = \Delta E + 0.24eV$$
(S4)

1.8.2. Computational parameters

The screening of optimal active sites, change of adsorption energy ΔE, charge distribution and density of state (DOS) for H chemisorption on the surfaces of various catalysts models were calculated using DFT in VASP package. The Perdew-Burke-Ernzerhof (PBE) functional within the generalized gradient approximation (GGA) was used to model the electronic

exchange correlation energy[1]. The projector augmented wave (PAW) method was used to describe the ionic cores. The cutoff energy was set to be 400 eV for the plane-wave expansion after testing a series of different cutoff energies. The K-points were set to be 4×4 ×1 for each model. Denser K-points of 6×6×1 was set to calculate differential charge distribution and Bader charge transfer serving as adsorption strength. Denser K-points of 12×12×1 was set to calculate the DOS serving as characterization of electronic structures[2].

1.8.3. Computational models

Based on different configurations of Ni_2P-Mo and Ni_2P-Mo-Fe demonstrated experimentally and theoretically (Supplementary Figure. 14-17), differential models were constructed to function as catalysts to chemisorb H simulating the first step of HER. Each model was periodic on both the x and y directions with an infinite large supercell sheet model. After optimization for all models, single H atom was added to all possible active sites on catalyst models to search for the best models acting as catalyst of HER.

2. Supplementary Figures S1-21

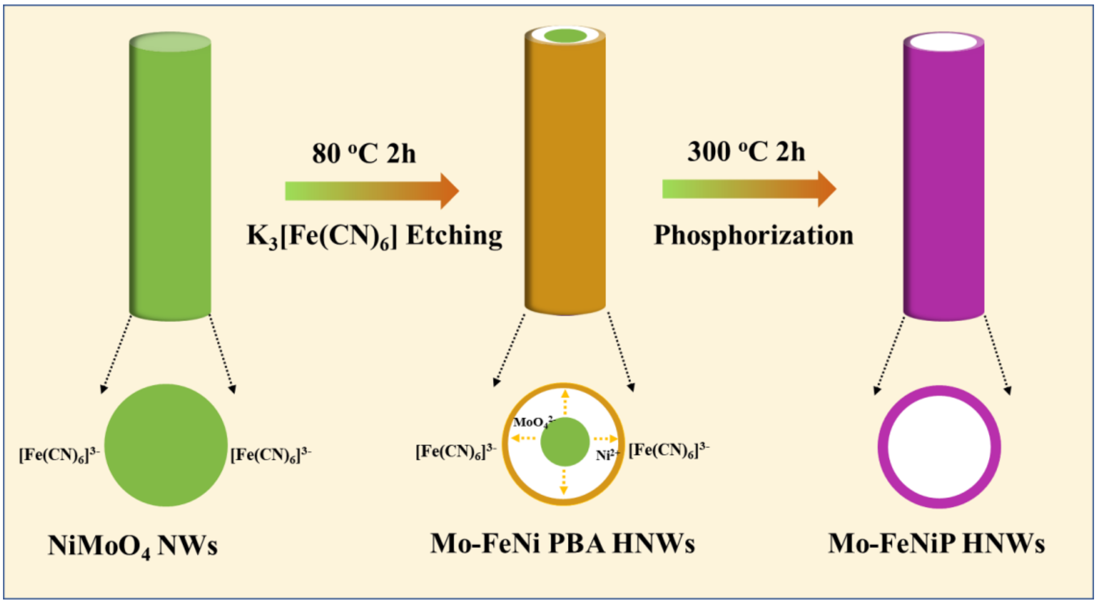

Figure S1. Schematic illustration for the formation process and conversion mechanism of Mo-FeNiP hollow nanotube structure.

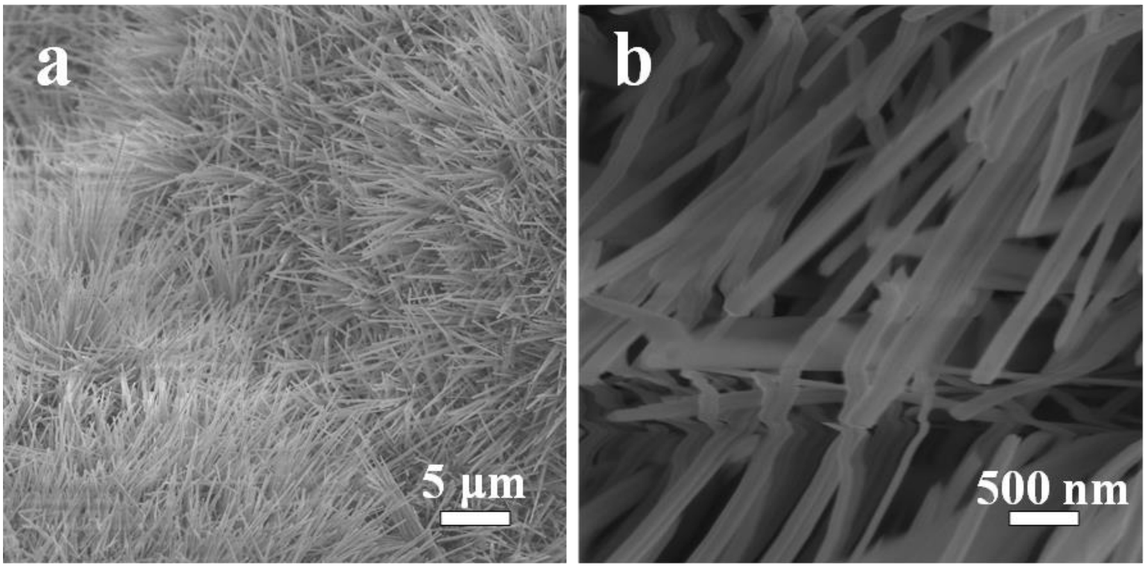

Figure S2. SEM images of $NiMoO_4$ NWs/NF.

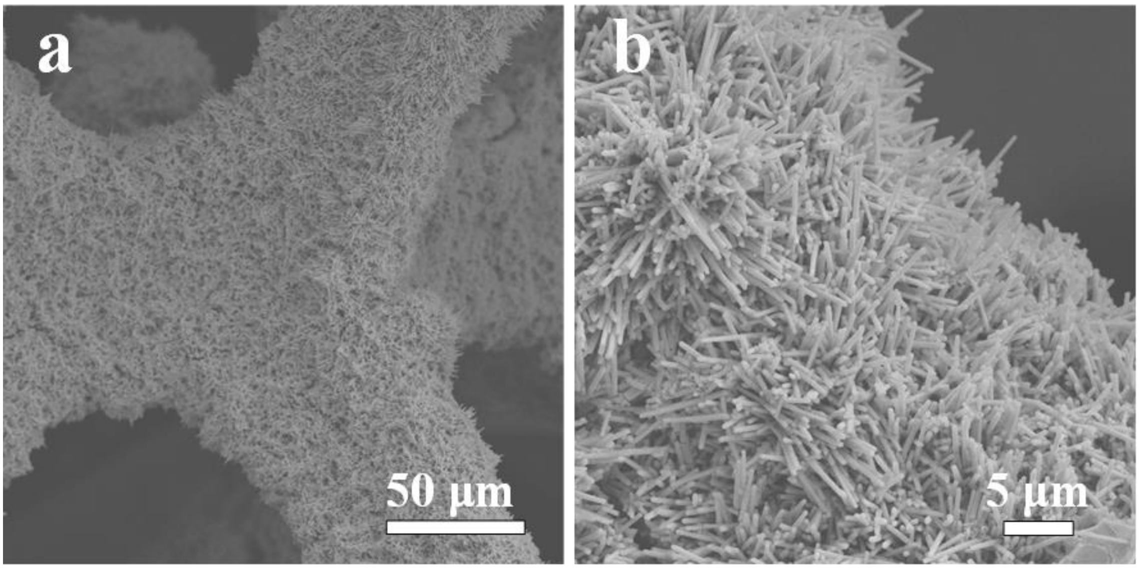

Figure S3. SEM images of Mo-FeNi PBA NTs/NF.

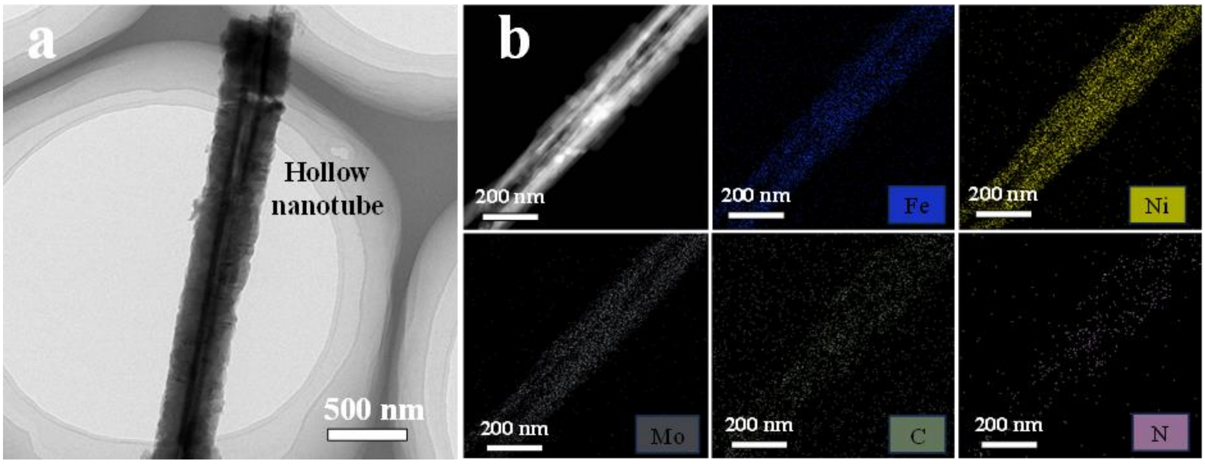

Figure S4. (a) TEM image and (b) EDS elemental mappings on Mo-FeNi PBA NTs.

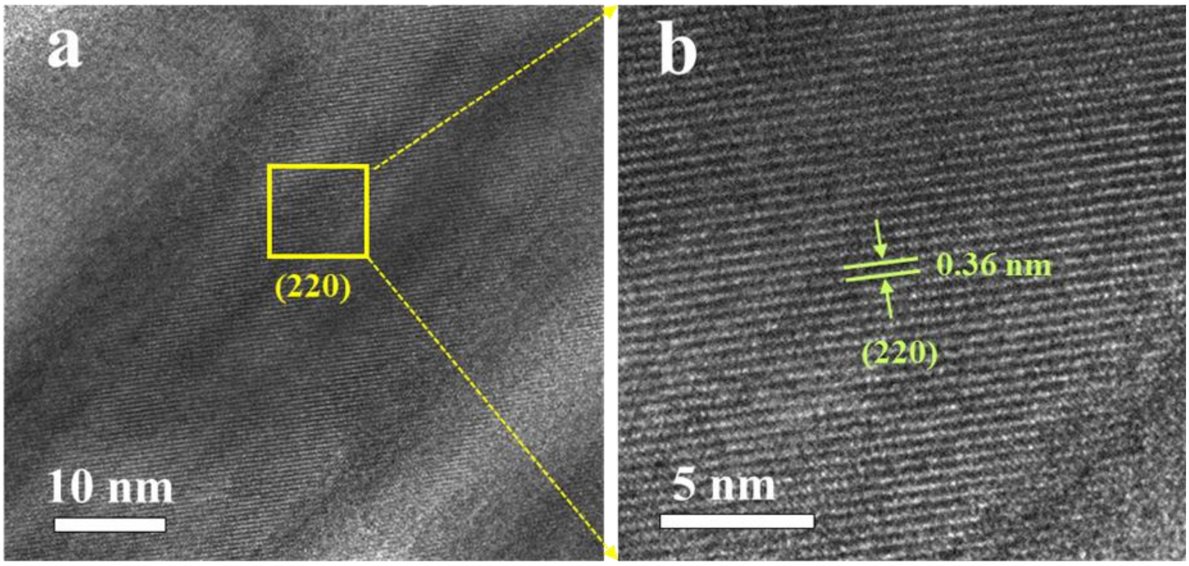

Figure S5. HRTEM images of Mo-FeNi PBA NTs.

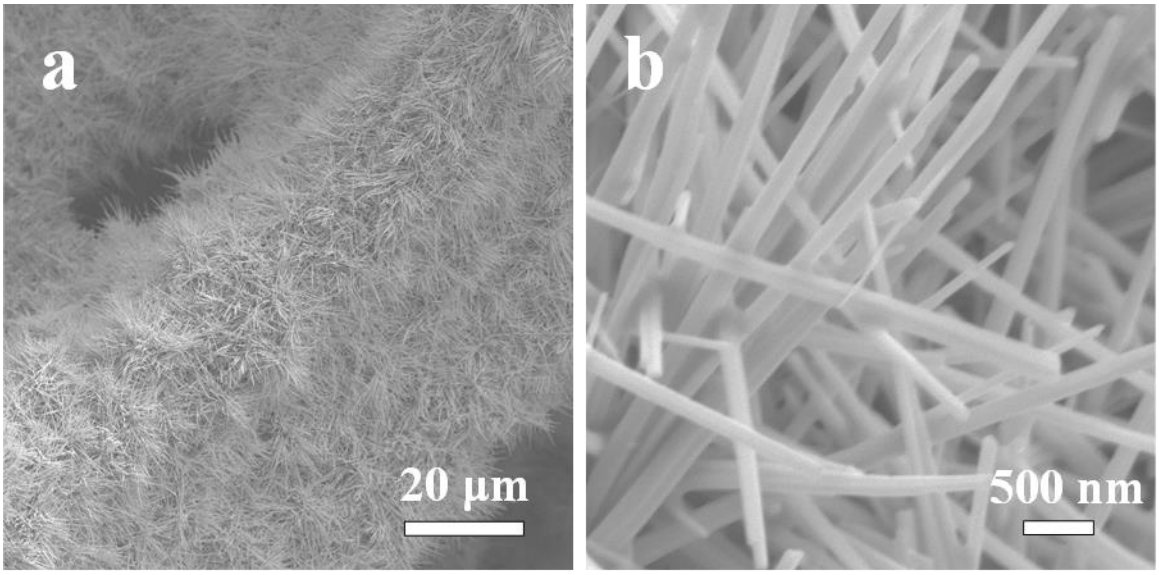

Figure S6. SEM images of NiMoP NWs/NF.

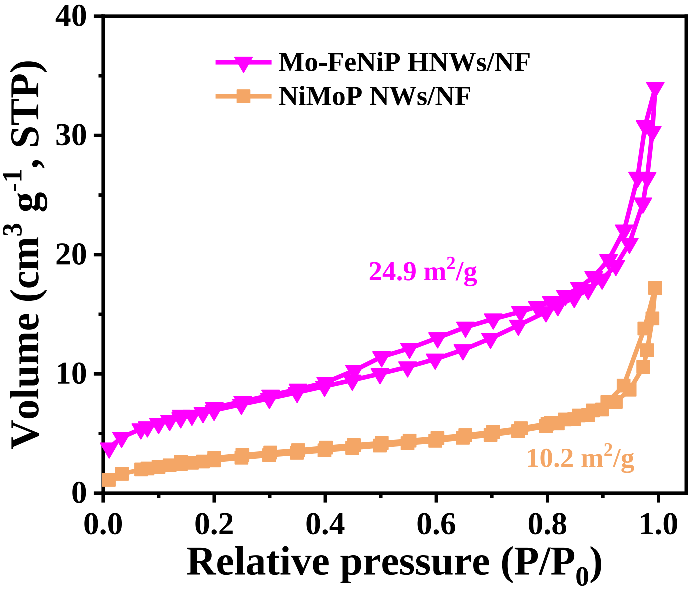

Figure S7. Nitrogen adsorption/desorption isotherms of NiMoP NWs/NF and Mo-FeNiP NTs/NF.

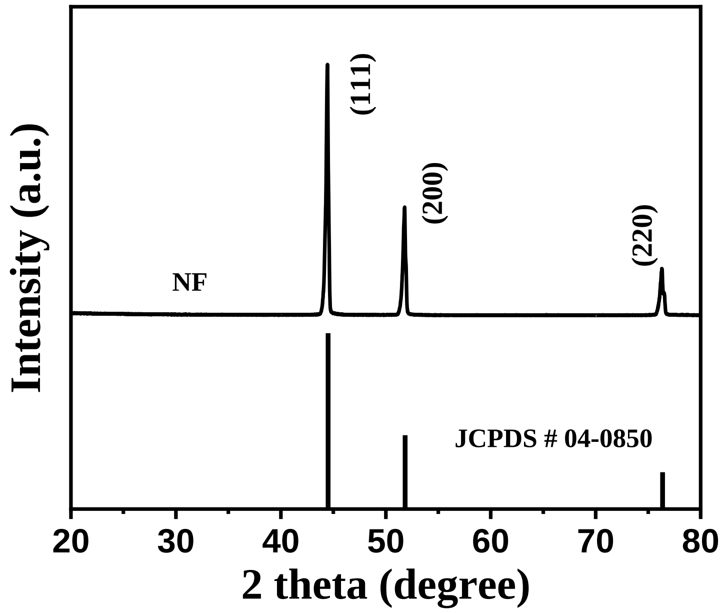

Figure S8. XRD pattern of NF substrate.

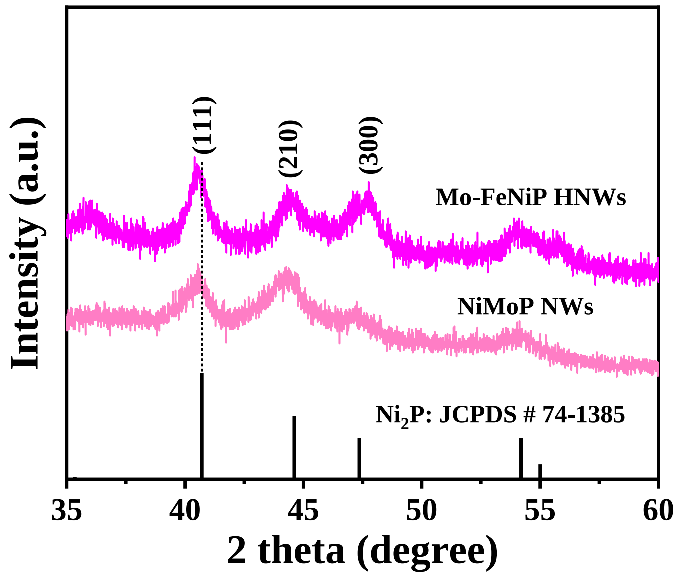

Figure S9. XRD patterns of NiMoP NWs and Mo-FeNiP NTs powders.

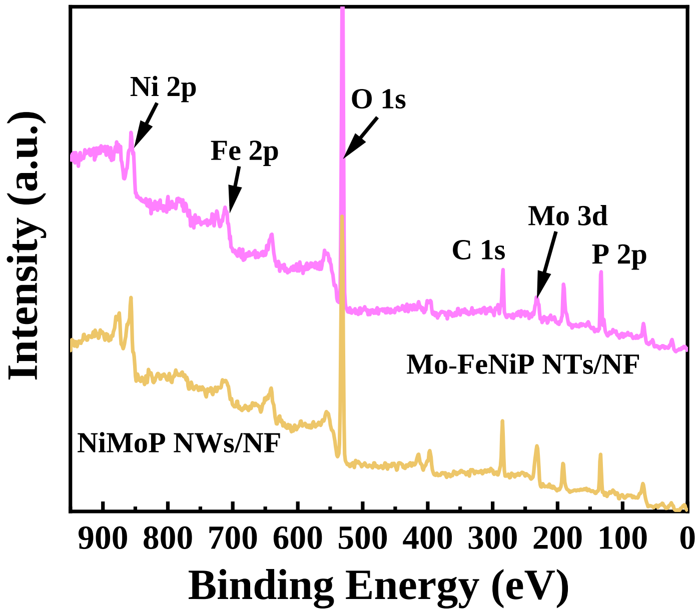

Figure S10. XPS surveys of NiMoP NWs/NF and Mo-FeNiP NTs/NF.

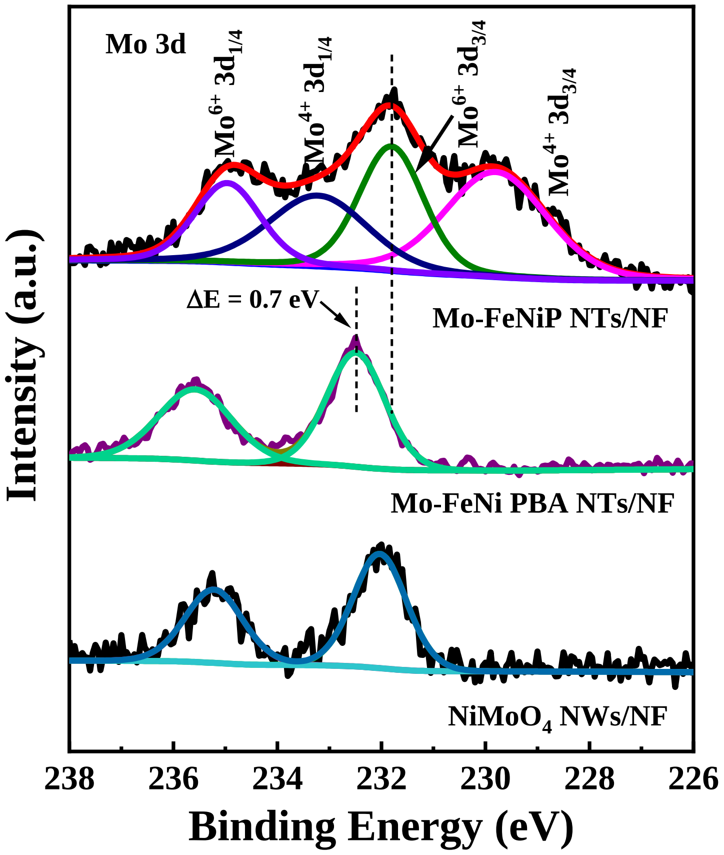

Figure S11. The comparison of XPS Mo 3d spectra for $NiMoO_4$ NWs/NF, Mo-FeNi PBA NTs/NF, and Mo-FeNiP NTs/NF.

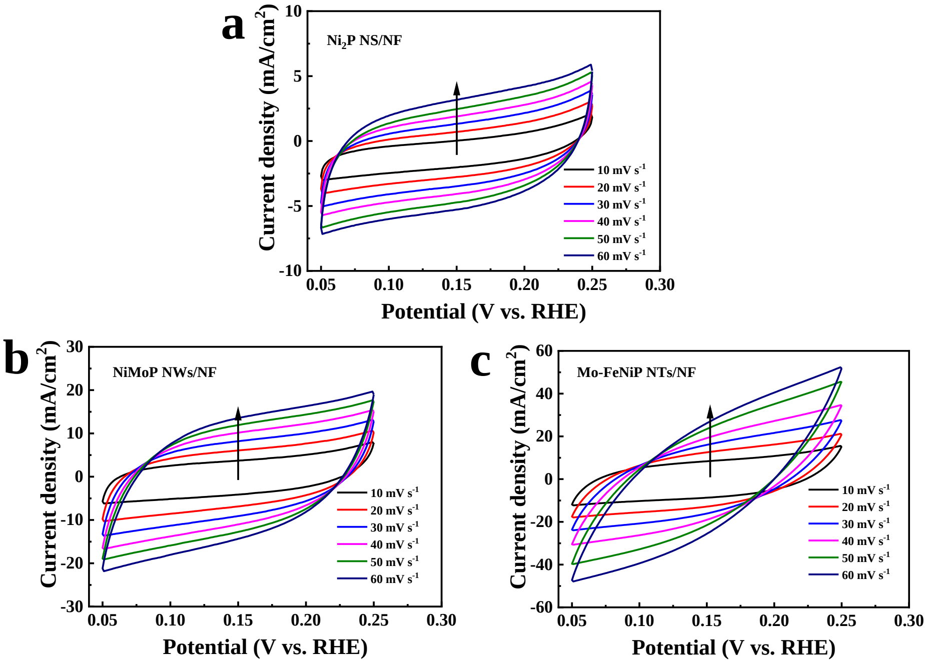

Figure S12. Voltammogram of (a) Ni_2P NS/NF, (b) NiMoP NWs/NF, and (c) Mo-FeNiP NTs/NF for HER.

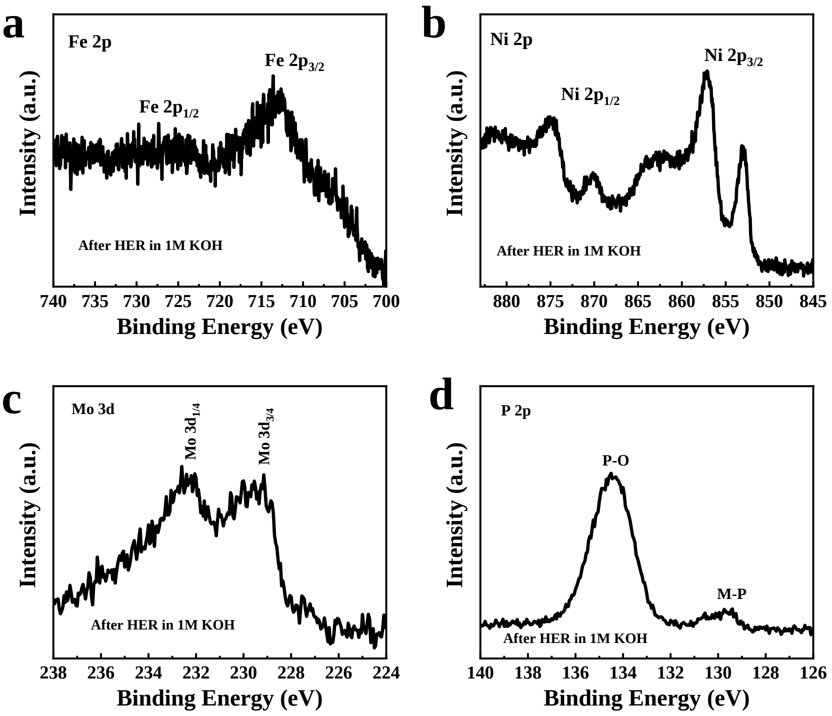

Figure S13. The XPS spectra of Mo-FeNiP NTs/NF after 3000 CV cycles for HER: (b) Fe 2p, (c) Ni 2p, (d) Mo 3d, and (f) P 2p.

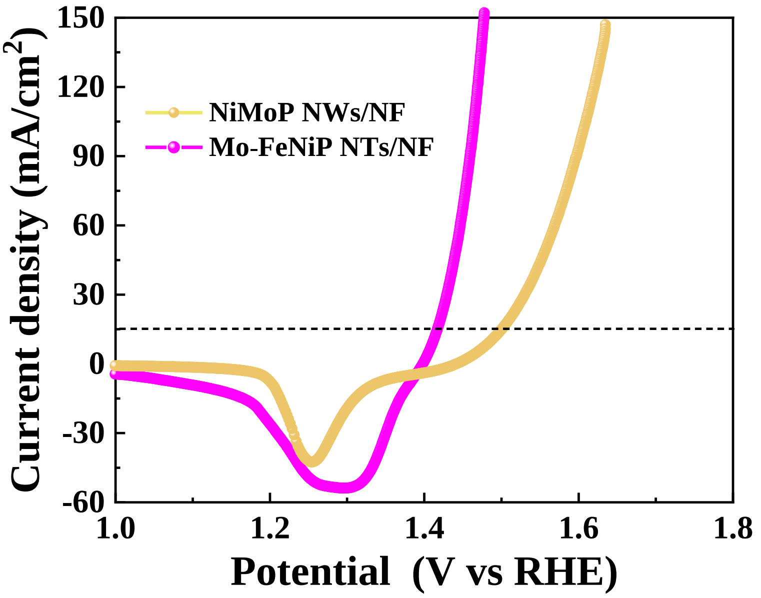

Figure S14. The OER polarization curves with a reverse scan for NiMoP NWs/NF and Mo-FeNiP NTs/NF.

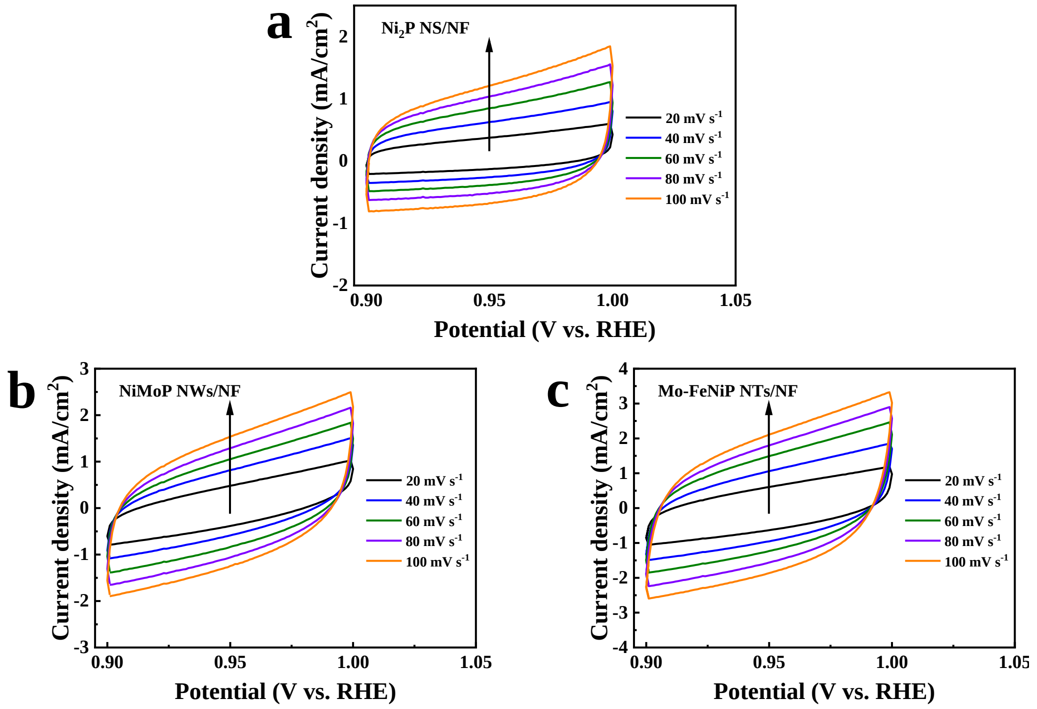

Figure S15. Voltammogram of (a) Ni_2P NS/NF, (b) NiMoP NWs/NF, and (c) Mo-FeNiP NTs/NF for OER.

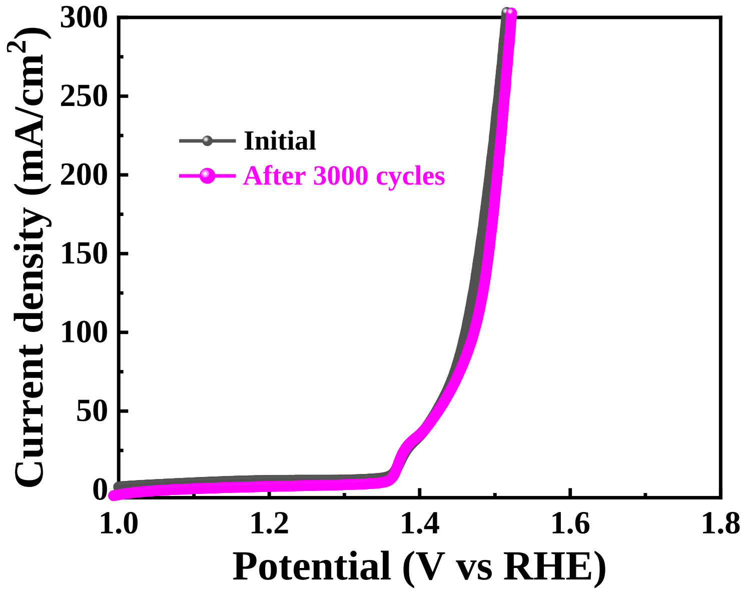

Figure S16. The polarization curves of Mo-FeNiP NTs/NF before and after 3000 CV cycles for OER.

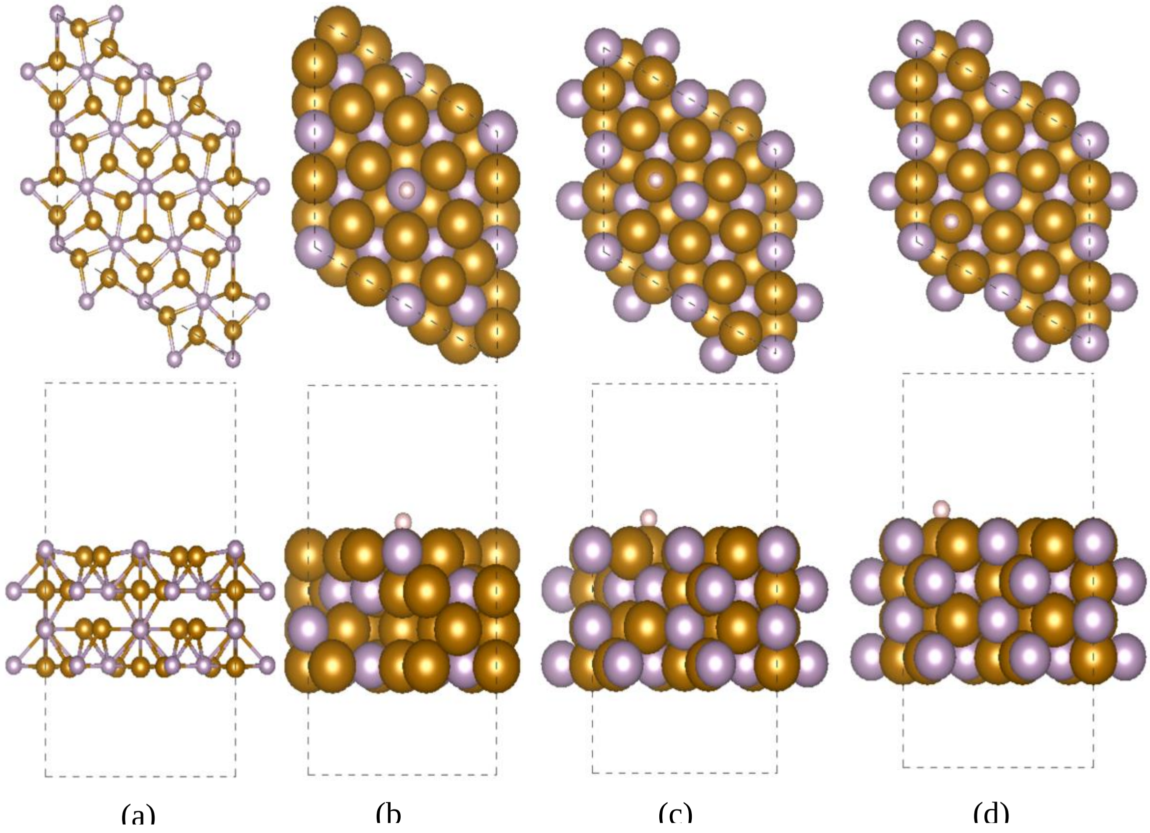

Figure S17. The optimal Fe_2P model: (a) Top view and side view of Fe_2P model, (b), (c), and (d) corresponding to the H chemisorption on different active sites, which are used to calculate the Gibbs free energy change before and after H chemisorption.

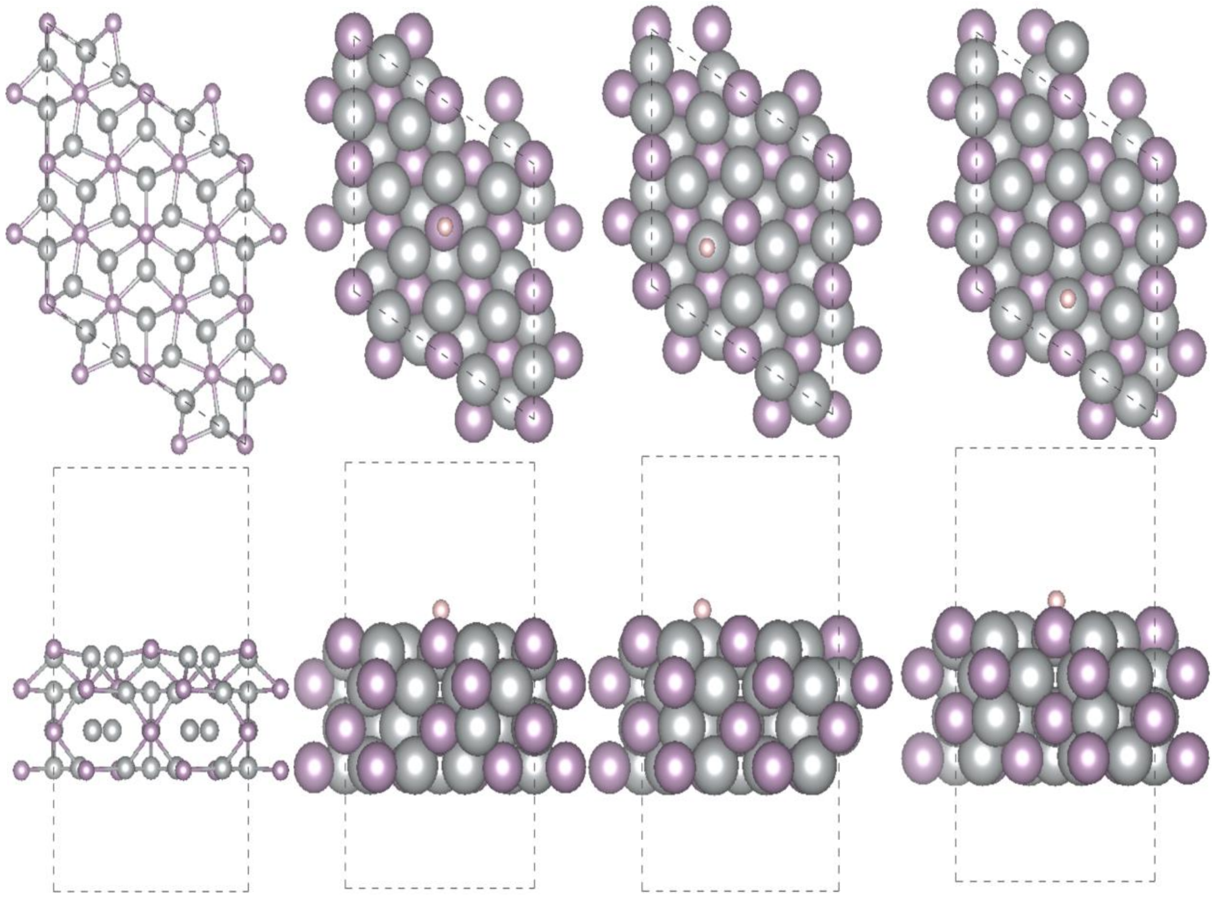

(a) (b (c) (d

Figure S18. The optimal Ni_2P model: (a) Top view and side view of Ni_2P model, (b), (c), and (d) corresponding to the H chemisorption on different active sites, which are used to calculate the Gibbs free energy change before and after H chemisorption.

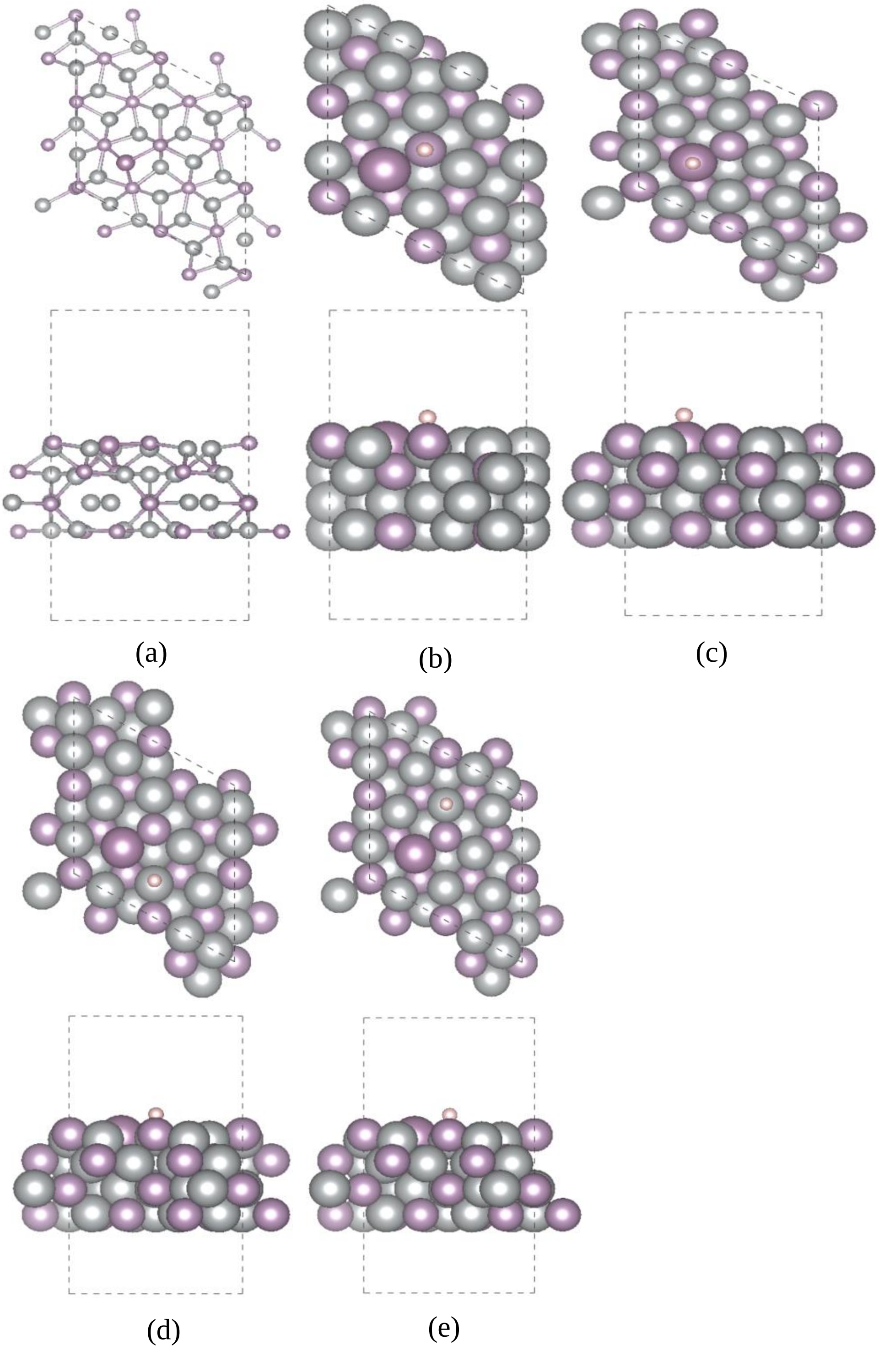

Figure S19. **The optimal Ni_2P-Mo model selected from two possible models: (a) Top view and side view of the Ni_2P-Mo model, (b), (c), (d) and (e) corresponding to the H chemisorption on different active sites, which are used to calculate the Gibbs free energy change before and after H chemisorption.**

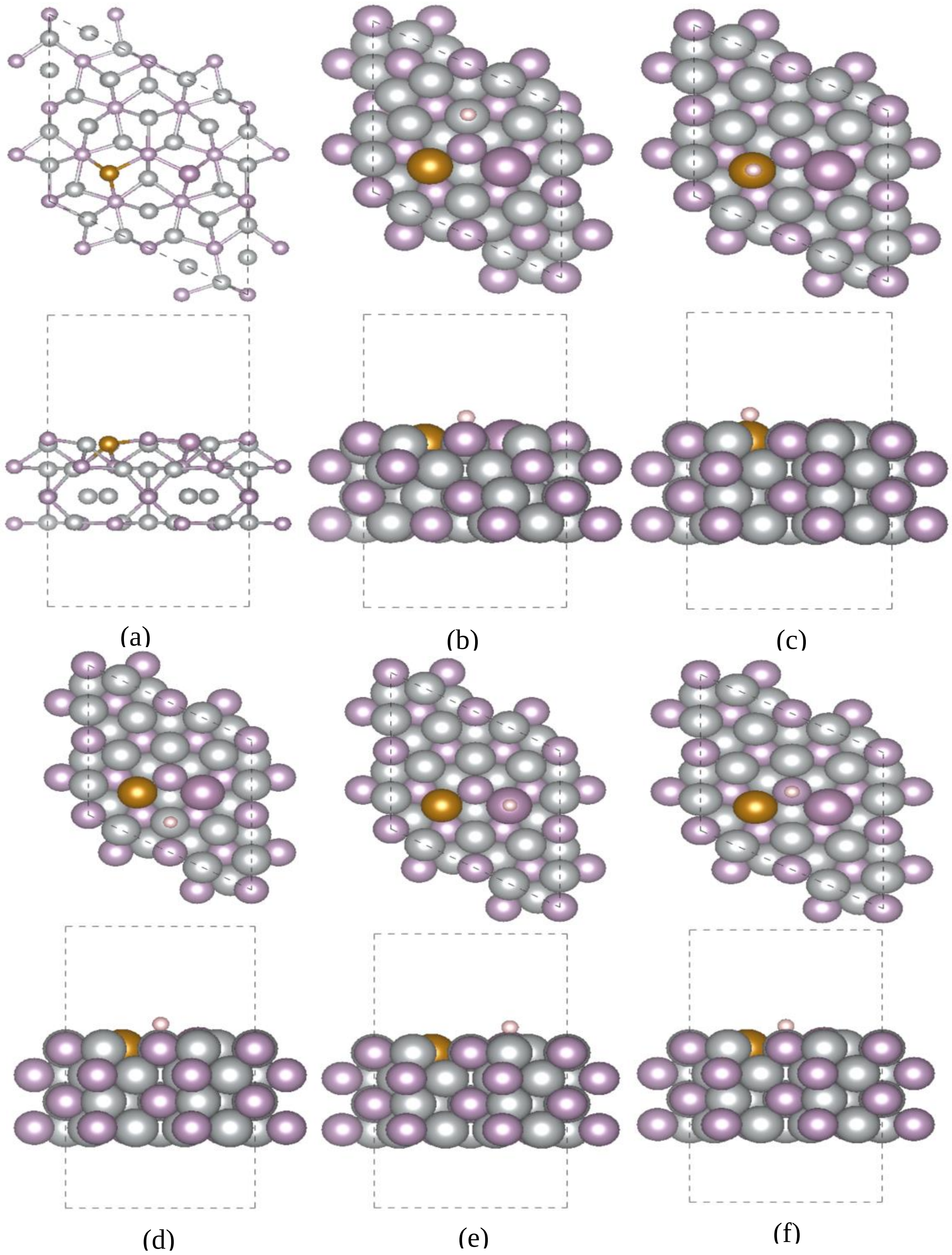

Figure S20. The optimal Ni_2P-Mo-Fe model selected from four possible models: (a) Top view and side view of Ni_2P-Mo-Fe model, (b), (c), (d), (e) and (f) corresponding to the H chemisorption on different active sites, which are used to calculate the Gibbs free energy change before and after H chemisorption.

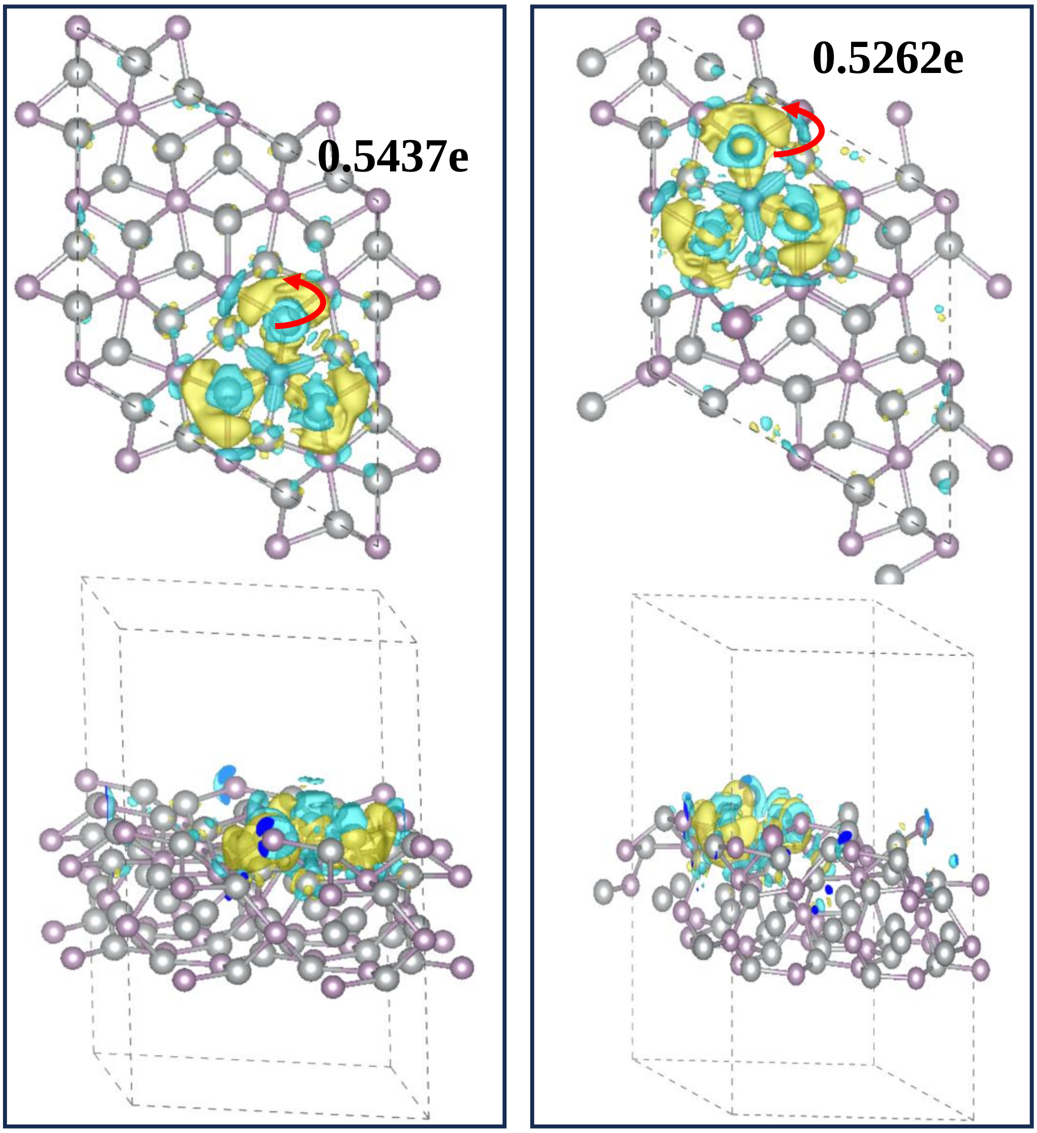

(a) (b)

Figure S21. Atomic structure and corresponding differential charge density and Bader charge transfer of the optimal a. Ni_2P, and b. Ni_2P-Mo. Blue color indicates positive charge and yellow color indicates negative values of electron quantities. The isosurface value is set to 0.004e/$Bohr^3$. The red arrow indicates the direction of charge transfer.

3. Supplementary Tables S1-3

Table S1. Comparison of electrochemical performances of Mo-FeNiP NTs for HER in 1.0 M KOH present work vs. literatures.

Catalyst	Substrate	Electrolyte	η_{10} (mV)	η_{100} (mV)	Ref
$NiS_{0.5}Se_{0.5}$	Ni foam	1 M KOH	70	~160	[3]
MoNiS	Ni foam	1 M KOH	98	200	[4]
N-NiMoS	Ni foam	1 M KOH	68	/	[5]
$CuMo_6S_8$/Cu	Cu foam	1 M KOH	172	>250	[6]
porous nickel	Ni foam	1 M KOH	101	203	[7]
A-NiCo LDH/NF	Ni foam	1 M KOH	~100	151	[8]
NC/Ni_3Mo_3N/NF	Ni foam	1 M KOH	44.6	/	[9]
N-MoO_2/Ni_3S_2NF	Ni foam	1 M KOH	225	300	[10]
N-WS_2/Ni_3FeN	Ni foam	1 M KOH	84	140	[11]
VS-Co_3S_4	GC	1 M KOH	63	~170	[12]
Ni–W	GC	1 M KOH	65	~190	[13]
Ni−MoO_2/NF	Ni foam	1 M KOH	49	153	[14]
MoS_2-Ni_3S_2 HNRs	NF	1 M KOH	98	191	[15]
GDY/MoO_3	Cu foam	0.1 M KOH	170	/	[16]
Ni/MoN/rNS	Ni sheet	1 M KOH	67	~200	[17]
MoP/Mo_2N	Ni foam	1 M KOH	89	190	[18]
NiP/poplar wood	poplar wood	1 M KOH	83	>150	[19]
MoNiFe alloy	Ni foam	1 M KOH	185	314	[20]
CoNi/$CoFe_2O_4$/NF	Ni foam	1 M KOH	82	189	[21]
Fe-Ni_2P@C/NF	Ni foam	1 M KOH	75	>200	[22]
$Ni_{12}P_5$–Fe_2P–NbP	Ni foam	1 M KOH	58	178	[23]
Cu-CoS_x/NF	Ni foam	1 M KOH	75	203	[24]
Ni_2P–$Ni_{12}P_5$	Ni foam	1 M KOH	76	147	[25]
Ni_xP NWs /NF	Ni foam	1 M KOH	71	153	[26]
$Ni_{2(1-x)}Mo_{2x}P$	Ni foam	1 M KOH	72	~160	[27]
$NiCo(OH)_x$-CuP	CF	1 M KOH	79	/	[28]
**Mo-FeNiP NTs**	**Ni foam**	**185**	**30.1**	**151.3**	**This work**

Table S2. Comparison of electrochemical performances of Mo-FeNiP NTs for OER in 1.0 M KOH present work vs. literatures.

**Catalyst**	**Substrate**	**Electrolyte**	**η_{10} (mV)**	**η_{100} (mV)**	**Ref**
NF@Fe_2-Ni_2P/C	Ni foam	1 M KOH	205	/	[29]
Fe doped CoCH/NF	Ni foam	1 M KOH	228	242	[30]
Ni_2P–Fe_2P/NF	Ni foam	1 M KOH	218	261	[31]
Fe_2P-Co_2P/CF	CF	1 M KOH	185	243	[32]
F-FeCoPv@IF	Fe foam	1 M KOH	/	280	[33]
CoNi/$CoFe_2O_4$/NF	Ni foam	1 M KOH	230	290	[34]
$CoMoN_x$	NP Au	1 M KOH	237	300	[35]
Ni/MoO_2@CN	Ni foam	1 M KOH	250	/	[36]
NF@NiFeLDH	Ni foam	1 M KOH	/	190	[37]
$N_{i3}Fe_{1-x}V_x$/CFP	CFP	1 M KOH	200	264	[38]
NiTe/NiS	Ni foam	1 M KOH	209	257	[39]
NiFe-MOF	Ni foam	1 M KOH	215	η_{50} =287	[40]
$FeNiCoCrMnS_2$	Ni foam	1 M KOH	199	246	[41]
FeMOFs-SO_3	Ni foam	1 M KOH	218	~245	[42]
FeOOH-NiBDC-NF	Ni foam	1 M KOH	278	/	[43]
Fe, P-$NiSe_2$	Ni foam	1 M KOH	/	266	[44]
FeCoCrNi alloy film	CC	1 M KOH	304	/	[45]
NiFeOOH	Ni foam	1 M KOH	210	~280	[46]
MoNiFe (oxy)hydroxide-27%	Carbon cloths	1 M KOH	242	290	[47]
NiFeB hydroxide	Carbon cloth	1 M KOH	209	252	[48]
FeP-CoP/NC	Ni foam	1 M KOH	281	392	[49]
FeCoNiMnRu/CNFs	CNFs	1 M KOH		308	[50]
NiFeMo oxyhydroxide	Ni foam	30% KOH, 85 °C	180	/	[51]
$Co_{3-x}Fe_xMo_3N$	Ni foil	1 M KOH	218	340	[52]
CoMn oxyhydroxide	Ni foam	1 M KOH	310	η_{200} =370	[53]
amorphous CoSe2	CF	1 M KOH	287	/	[54]
**Mo-FeNiP NTs**	**Ni foam**	**1 M KOH**	**182.5**	**151.3**	**This work**

Table S3. Comparison of reported bifunctional electrocatalysts with Mo-FeNiP NTs for overall water splitting performances present work vs. literatures.

Catalysts	Overpotential @10 mA cm^{-2} (mV)		Cell Voltage @10 mA cm^{-2} (V)	Stability (h)	Electrolyte	Ref.
	HER	OER				
Ni_2P@$FePO_x$	75	205	1.51	100	1 M KOH	[55]
$NiFeO_x$@NiFeP	200	220	1.65	600	1 M NaOH	[56]
Co_2P/Ni_2P-2%Mo	50	319(50)	1.56	50	1 M KOH	[57]
CoP/Co_2P	103	317	1.65	8	1 M KOH	[58]
CoP–FeP	71	250	1.55	–	1 M KOH	[59]
FeP_2–NiP_2@PC	179	248	1.7	–	1 M KOH	[60]
MoP/Ni_2P/NF	75	309(20)	1.55	15	1 M KOH	[61]
$NiCo_2O_4/Ni_2P$	45	250	1.59	50	1 M KOH	[62]
NiFe LDH/NiCoP	120	220	1.57	100	1 M KOH	[63]
CoP-Co_2P@PC/PG	39	272(20)	1.567	5000 CV	1 M KOH	[64]
Ni_2P@G	103	275(20)	1.51	24	1 M KOH	[65]
NiCoFeP/C	149	270	1.6	18	1 M KOH	[66]
NiP/NiFeP/C	87	250	1.53	20	1 M KOH	[67]
FeNiP/PG	173	229	1.63	20	1 M KOH	[68]
NiCoP@NCNA/NF	37	305	1.57	–	1 M KOH	[69]
Ni_2P/Ni_3S_2/NF	80	210	1.5	36	1 M KOH	[70]
Fe-Ni_2P/MoS_x/NF	112	246	1.61	35	1 M KOH	[71]
NiS/Ni_2P/CC	111	265	1.67	10	1 M KOH	[72]
$Fe_{0.29}Co_{0.71}P$/NF	74	251(50)	1.59	–	1 M KOH	[73]
NiCoP@NCNA/NF	37	305(50)	1.56	22	1 M KOH	[74]
Co_4Ni_1P NTs	129	245	1.59	50	1 M KOH	[75]
Co_2P/CNTs	132	292	1.53	50	1 M KOH	[76]

NiCoP/rGO	59	270	1.59	75	1 M KOH	[77]
Ni–V-based TMIC	64(Ni_3N-VN)	220(Ni_2P-VP_2)	1.51	10	1 M KOH	[78]
V/NF	176	292	1.74	24	1 M KOH	[79]
S-CoWP@(S,N)-C	67	280	1.65	15	1 M KOH	[80]
Nix$Co_{3-x}O_4$/NiCo/NiCoO_x	155	337	1.75	10	1 M KOH	[81]
FeNi–P/NCN	190	240	1.54	20	1 M KOH	[82]
a-CoMoP_x/CF	59	305	1.581	100	1 M KOH	[83]
Ni_2P-$NiSe_2$/CC	89	250	1.56	90	1 M KOH	[84]
CoP@FeCoP/NC	141	238	1.68	20	1 M KOH	[85]
Co-P-B-5	145	290	1.64	-	1 M NaOH	[86]
$Fe_{0.4}Co_{0.3}Ni_{0.3}$	184	175	1.62	50	1 M KOH	[87]
(CoNiP@NiFe LDHs	68	255	1.59	50	1 M KOH	[88]
**Mo-FeNiP NTs/NF**	**30.1**	**182.5**	**1.47**	**100**	1 M KOH	**This work**

Table S4. The different adsorption energy change (ΔE, unit eV) at ground state and corresponding Gibbs free energy change (ΔG, unit eV) at ambient condition (298K, 0.1MPa) upon adsorbing a H molecule on possible active sites of selected optimal models.

**Models**	***Active site***	**Δ*E* (eV)**	**Δ*G*(eV)**
**Fe_2P**	1	-0.13068	0.109315
	2	-1.31923	-1.07923
	3	-1.39842	-1.15842
**Ni_2P**	1	0.15045	0.39045
	2	0.238466	0.478466
	3	-0.30067	-0.06067
**Ni_2P-Mo-1**	1	0.329353	0.569353
	2	-0.28393	-0.04393
	3	-0.5713	-0.3313
	4	-0.2668	-0.0268
**Ni_2P-Mo-2**	1	-0.00746	0.232542

	2	-0.57248	-0.33248
	3	-0.28389	-0.04389
	4	-0.57111	-0.33111
**Ni_2P-Mo-Fe-1**	1	-0.24768	-0.00768036
	2	-0.37635	-0.13635228
	3	-0.5644	-0.32440077
	4	-0.31281	-0.07281161
	5	0.229873	0.46987287
**Ni_2P-Mo-Fe-2**	1	0.080913	0.320913
	2	-0.57322	-0.33321642
	3	-0.30977	-0.06977237
	4	-0.57329	-0.3332893
	5	-0.5488	-0.30880003
**Ni_2P-Mo-Fe-3**	1	-0.37616	-0.13616235
	2	-0.56456	-0.32455568
	3	-0.31218	-0.0721824
	4	0.230265	0.47026542
	5	-0.24768	-0.00768468
**Ni_2P-Mo-Fe-4**	1	-0.34927	-0.10927445
	2	-0.60227	-0.36227144
	3	-0.27632	-0.03631918
	4	0.257662	0.49766187
	5	-0.2564	-0.01639876

4. References

[1] Y. Gao, Z. Li, P. Wang, W. Cui, X. Wang, Y. Yang, F. Gao, M. Zhang, J. Gan, C. Li, Y. Liu, X. Wang, F. Qi, J. Zhang, X. Han, W. Du, H. Pan, Z. Xia, Adv. Funct. Mater. 2023, 33, 2305610.

[2] Y. Gao, J. Zhang, X. Luo, Y. Wan, Z. Zhao, X. Han, Z. Xia, Nano Energy 2020, 72, 104666.

[3] Y. Wang, X. Li, M. Zhang, Y. Zhou, D. Rao, C. Zhong, J. Zhang, X. Han, W. Hu, Y. Zhang, K. Zaghib, Y. Wang, Y. Deng, Adv. Mater. 2020, 32, 2000231.

[4] Y. Cheng, H. Guo, L. Zhang, M. Wang, J. Zhou, T. Qian, C. Yan, Adv. Funct. Mater. 2023, 33, 2208718.

[5] C. Huang, L. Yu, W. Zhang, Q. Xiao, J. Zhou, Y. Zhang, P. An, J. Zhang, Y. Yu, Applied catalysis. B, Environmental 2020, 276, 119137.

[6] H. Liu, R. Xie, Y. Luo, Z. Cui, Q. Yu, Z. Gao, Z. Zhang, F. Yang, X. Kang, S. Ge, S. Li, X. Gao, G. Chai, L. Liu, B. Liu, Nat. Commun. 2022, 13, 6382.

[7] F. Xiao, L. Li, W. Cui, Y. Zhang, C. Zhan, W. Xiao, J. Power Sources 2023, 556, 11.

[8] H. Yang, Z. Chen, P. Guo, B. Fei, R. Wu, Applied Catalysis B: Environmental 2020, 261, 118240.

[9] Y. Chen, J. Yu, J. Jia, F. Liu, Y. Zhang, G. Xiong, R. Zhang, R. Yang, D. Sun, H. Liu, W. Zhou, Applied Catalysis B: Environmental 2020, 272, 118956.

[10] L. Wang, J. Cao, C. Lei, Q. Dai, B. Yang, Z. Li, X. Zhang, C. Yuan, L. Lei, Y. Hou, ACS Appl. Mater. Interfaces 2019, 11, 27743.

[11] J. Zeng, L. Zhang, Q. Zhou, L. Liao, Y. Qi, H. Zhou, D. Li, F. Cai, H. Wang, D. Tang, F. Yu, Small 2022, 18, 2104624.
[12] C. Zhang, Y. Shi, Y. Yu, Y. Du, B. Zhang, ACS Catal. 2018, 8, 8077.
[13] H. Wu, L. Kong, Y. Ji, J. Yan, Y. Ding, Y. Li, S. T. Lee, S. F. Liu, Adv. Mater. Interfaces 2019, 6, 1900308.
[14] J. Ren, X. Wu, T. Liu, L. Chen, R. Hao, Y. Song, Y. Liu, Z. Yuan, Appl. Catal. B-Environ. 2022, 317, 14.
[15] Y. Yang, K. Zhang, H. Lin, X. Li, H. C. Chan, L. Yang, Q. Gao, ACS Catal. 2017, 7, 2357.
[16] Y. Yao, Y. Zhu, C. Pan, C. Wang, S. Hu, W. Xiao, X. Chi, Y. Fang, J. Yang, H. Deng, S. Xiao, J. Li, Z. Luo, Y. Guo, J. Am. Chem. Soc. 2021, 143, 8720.
[17] Y. Chen, Y. Wang, J. Yu, G. Xiong, H. Niu, Y. Li, D. Sun, X. Zhang, H. Liu, W. Zhou, Adv. Sci. 2022, 9, 2105869.
[18] Y. Gu, A. Wu, Y. Jiao, H. Zheng, X. Wang, Y. Xie, L. Wang, C. Tian, H. Fu, Angewandte Chemie International Edition 2021, 60, 6673.
[19] B. Hui, K. Zhang, Y. Xia, C. Zhou, Electrochim. Acta 2020, 330, 135274.
[20] N. S. Gultom, T. Chen, M. Z. Silitonga, D. Kuo, Applied Catalysis B: Environmental 2023, 322, 122103.
[21] S. Li, S. Sirisomboonchai, A. Yoshida, X. An, X. Hao, A. Abudula, G. Guan, J. Mater. Chem. A 2018, 6, 10.
[22] D. Li, Z. Li, R. Zou, G. Shi, Y. Huang, W. Yang, W. Yang, C. Liu, X. Peng, Applied Catalysis B: Environmental 2022, 307, 121170.
[23] S. Wen, G. Chen, W. Chen, M. Li, B. Ouyang, X. Wang, D. Chen, T. Gong, X. Zhang, J. Huang, K. K. Ostrikov, J. Mater. Chem. A 2021, 9, 9918.
[24] H. He, L. Zeng, X. Peng, Z. Liu, D. Wang, B. Yang, Z. Li, L. Lei, S. Wang, Y. Hou, Chem. Eng. J. 2023, 451, 138628.
[25] Z. Wang, S. Wang, L. Ma, Y. Guo, J. Sun, N. Zhang, R. Jiang, Small 2021, 17, 2006770.
[26] Y. Wang, L. Liu, X. Zhang, F. Yan, C. Zhu, Y. Chen, J. Mater. Chem. A 2019, 7, 8.
[27] L. Yu, I. K. Mishra, Y. Xie, H. Zhou, J. Sun, J. Zhou, Y. Ni, D. Luo, F. Yu, Y. Yu, S. Chen, Z. Ren, Nano Energy 2018, 53, 492.
[28] F. Meng, P. Ren, Z. Li, R. Li, Y. Li, H. Zhang, A. Liu, V. Kondratiev, O. Levin, J. Zhang, M. An, P. Yang, J. Alloys Compd. 2023, 946, 169465.
[29] H. Sun, Y. Min, W. Yang, Y. Lian, L. Lin, K. Feng, Z. Deng, M. Chen, J. Zhong, L. Xu, Y. Peng, ACS Catal. 2019, 9, 8882.
[30] L. Hui, Y. Xue, D. Jia, H. Yu, C. Zhang, Y. Li, Adv. Energy Mater. 2018, 8, 1800175.
[31] L. Wu, L. Yu, F. Zhang, B. McElhenny, D. Luo, A. Karim, S. Chen, Z. Ren, Adv. Funct. Mater. 2021, 31, 2006484.
[32] X. Liu, Y. Yao, H. Zhang, L. Pan, C. Shi, X. Zhang, Z. Huang, J. Zou, ACS Sustain. Chem. Eng. 2020, 8, 17828.
[33] J. Zhu, J. Chi, T. Cui, L. Guo, S. Wu, B. Li, J. Lai, L. Wang, Appl. Catal. B 2023, 328, 122487.
[34] S. Li, S. Sirisomboonchai, A. Yoshida, X. An, X. Hao, A. Abudula, G. Guan, Journal of materials chemistry. A, Materials for energy and sustainability 2018, 6, 19221.
[35] R. Q. Yao, H. Shi, W. B. Wan, Z. Wen, X. Y. Lang, Q. Jiang, Adv. Mater. 2020, 32, 1907214.
[36] G. Qian, J. Chen, T. Yu, J. Liu, L. Luo, S. Yin, Nano-Micro Lett. 2022, 14, 20.
[37] X. Li, C. Liu, Z. Fang, L. Xu, C. Lu, W. Hou, Small 2022, 18, 2104354.
[38] J. Jiang, F. Sun, S. Zhou, W. Hu, H. Zhang, J. Dong, Z. Jiang, J. Zhao, J. Li, W. Yan, M. Wang, Nat. Commun. 2018, 9, 2885.

[39] Z. Xue, X. Li, Q. Liu, M. Cai, K. Liu, M. Liu, Z. Ke, X. Liu, G. Li, Adv. Mater. 2019, 31, e1900430.
[40] J. Zhou, Z. Han, X. Wang, H. Gai, Z. Chen, T. Guo, X. Hou, L. Xu, X. Hu, M. Huang, S. V. Levchenko, H. Jiang, Adv. Funct. Mater. 2021, 31, 2102066.
[41] T. X. Nguyen, Y. Su, C. Lin, J. Ting, Adv. Funct. Mater. 2021, 31, 2106229.
[42] K. Feng, D. Zhang, F. Liu, H. Li, J. Xu, Y. Xia, Y. Li, H. Lin, S. Wang, M. Shao, Z. Kang, J. Zhong, Adv. Energy Mater. 2020, 10, 2000184.
[43] R. Fan, J. Xie, H. Liu, H. Wang, M. Li, N. Yu, R. Luan, Y. Chai, B. Dong, Chem. Eng. J. 2022, 431, 134040.
[44] J. Chang, G. Wang, Z. Yang, B. Li, Q. Wang, R. Kuliiev, N. Orlovskaya, M. Gu, Y. Du, G. Wang, Y. Yang, Adv. Mater. 2021, 33, 2101425.
[45] N. Zhang, X. Feng, D. Rao, X. Deng, L. Cai, B. Qiu, R. Long, Y. Xiong, Y. Lu, Y. Chai, Nat. Commun. 2020, 11, 4066.
[46] C. Jia, C. Zhen, L. Yin, H. Zhu, P. Du, A. Han, G. Liu, H. Cheng, Nano Energy 2023, 106, 108044.
[47] Z. He, J. Zhang, Z. Gong, H. Lei, D. Zhou, N. Zhang, W. Mai, S. Zhao, Y. Chen, Nat. Commun. 2022, 13.
[48] Y. Bai, Y. Wu, X. Zhou, Y. Ye, K. Nie, J. Wang, M. Xie, Z. Zhang, Z. Liu, T. Cheng, C. Gao, Nat. Commun. 2022, 13, 6094.
[49] X. Yan, J. Biemolt, K. Zhao, Y. Zhao, X. Cao, Y. Yang, X. Wu, G. Rothenberg, N. Yan, Nat. Commun. 2021, 12, 4143.
[50] J. Hao, Z. Zhuang, K. Cao, G. Gao, C. Wang, F. Lai, S. Lu, P. Ma, W. Dong, T. Liu, M. Du, H. Zhu, Nat. Commun. 2022, 13, 2662.
[51] B. Zhang, L. Wang, Z. Cao, S. M. Kozlov, F. P. García De Arquer, C. T. Dinh, J. Li, Z. Wang, X. Zheng, L. Zhang, Y. Wen, O. Voznyy, R. Comin, P. De Luna, T. Regier, W. Bi, E. E. Alp, C. Pao, L. Zheng, Y. Hu, Y. Ji, Y. Li, Y. Zhang, L. Cavallo, H. Peng, E. H. Sargent, Nat. Catal. 2020, 3, 985.
[52] C. Zhong, J. Zhang, L. Zhang, Y. Tu, H. Song, L. Du, Z. Cui, ACS Energy Lett. 2023, 8, 1455.
[53] Y. Devi, P. Huang, W. Chen, R. Jhang, C. Chen, ACS Appl. Mater. Interfaces 2023, 15, 9231.
[54] R. Li, Q. Li, L. Xiao, X. Bai, S. Ji, J. Zhang, M. An, P. Yang, Mater. Today Energy 2021, 22, 100882.
[55] F. S. Zhang, J. W. Wang, J. Luo, R. R. Liu, Z. M. Zhang, C. T. He, T. B. Lu, Chem. Sci. 2018, 9, 1375.
[56] F. Hu, Y. Zhang, X. Shen, J. Tao, X. Yang, Y. Xiong, Z. Peng, J. Power Sources 2019, 428, 76.
[57] H. Liu, M. Jin, D. Zhan, J. Wang, X. Cai, Y. Qiu, L. Lai, Applied catalysis. B, Environmental 2020, 272, 118951.
[58] L. Chen, Y. Zhang, H. Wang, Y. Wang, D. Li, C. Duan, Nanoscale 2018, 10, 21019.
[59] Z. Niu, C. Qiu, J. Jiang, L. Ai, ACS Sustain. Chem. Eng. 2019, 7, 2335.
[60] P. Ji, H. Jin, H. Xia, X. Luo, J. Zhu, Z. Pu, S. Mu, ACS Appl. Mater. Interfaces 2020, 12, 727.
[61] C. Du, M. Shang, J. Mao, W. Song, Journal of materials chemistry. A, Materials for energy and sustainability 2017, 5, 15940.
[62] L. Wang, C. Gu, X. Ge, J. Zhang, H. Zhu, J. Tu, Adv. Mater. Interfaces 2017, 4, 1700481.
[63] H. Zhang, X. Li, A. Hähnel, V. Naumann, C. Lin, S. Azimi, S. L. Schweizer, A. W. Maijenburg, R. B. Wehrspohn, Adv. Funct. Mater. 2018, 28, 1706847.

[64] J. Yang, D. Guo, S. Zhao, Y. Lin, R. Yang, D. Xu, N. Shi, X. Zhang, L. Lu, Y. Q. Lan, J. Bao, M. Han, Small 2019, 15, e1804546.
[65] H. Mou, J. Wang, D. Yu, D. Zhang, F. Lu, L. Chen, D. Wang, T. Mu, Journal of materials chemistry. A, Materials for energy and sustainability 2019, 7, 13455.
[66] X. Wei, Y. Zhang, H. He, L. Peng, S. Xiao, S. Yao, P. Xiao, Chemical communications (Cambridge, England) 2019, 55, 10896.
[67] B. Weng, X. Wang, C. R. Grice, F. Xu, Y. Yan, Journal of materials chemistry. A, Materials for energy and sustainability 2019, 7, 7168.
[68] F. Bu, W. Chen, M. F. Aly Aboud, I. Shakir, J. Gu, Y. Xu, Journal of materials chemistry. A, Materials for energy and sustainability 2019, 7, 14526.
[69] B. Cao, Y. Cheng, M. Hu, P. Jing, Z. Ma, B. Liu, R. Gao, J. Zhang, Adv. Funct. Mater. 2019, 29, 1906316.
[70] L. Zeng, K. Sun, X. Wang, Y. Liu, Y. Pan, Z. Liu, D. Cao, Y. Song, S. Liu, C. Liu, Nano Energy 2018, 51, 26.
[71] X. Zhang, C. Liang, X. Qu, Y. Ren, J. Yin, W. Wang, M. Yang, W. Huang, X. Dong, Adv. Mater. Interfaces 2020, 7, 1901926.
[72] X. Xiao, D. Huang, Y. Fu, M. Wen, X. Jiang, X. Lv, M. Li, L. Gao, S. Liu, M. Wang, C. Zhao, Y. Shen, ACS Appl. Mater. Interfaces 2018, 10, 4689.
[73] H. Feng, L. Tang, G. Zeng, J. Yu, Y. Deng, Y. Zhou, J. Wang, C. Feng, T. Luo, B. Shao, Nano Energy 2020, 67, 104174.
[74] B. Cao, Y. Cheng, M. Hu, P. Jing, Z. Ma, B. Liu, R. Gao, J. Zhang, Adv. Funct. Mater. 2019, 29, 1906316.
[75] L. Yan, L. Cao, P. Dai, X. Gu, D. Liu, L. Li, Y. Wang, X. Zhao, Adv. Funct. Mater. 2017, 1703455.
[76] D. Das, K. K. Nanda, Nano Energy 2016, 30, 303.
[77] J. Li, M. Yan, X. Zhou, Z. Huang, Z. Xia, C. Chang, Y. Ma, Y. Qu, Adv. Funct. Mater. 2016, 26, 6785.
[78] H. Yan, Y. Xie, A. Wu, Z. Cai, L. Wang, C. Tian, X. Zhang, H. Fu, Adv. Mater. 2019, 31, 1901174.
[79] Y. Yu, P. Li, X. Wang, W. Gao, Z. Shen, Y. Zhu, S. Yang, W. Song, K. Ding, Nanoscale 2016, 8, 10731.
[80] B. Weng, C. R. Grice, W. Meng, L. Guan, F. Xu, Y. Yu, C. Wang, D. Zhao, Y. Yan, ACS Energy Lett. 2018, 3, 1434.
[81] X. Yan, K. Li, L. Lyu, F. Song, J. He, D. Niu, L. Liu, X. Hu, X. Chen, ACS Appl. Mater. Interfaces 2016, 8, 3208.
[82] Y. Wu, X. Tao, Y. Qing, H. Xu, F. Yang, S. Luo, C. Tian, M. Liu, X. Lu, Adv. Mater. 2019, 31, 1900178.
[83] H. Huang, A. Cho, S. Kim, H. Jun, A. Lee, J. W. Han, J. Lee, Adv. Funct. Mater. 2020, 30, 2003889.
[84] L. Yang, L. Huang, Y. Yao, L. Jiao, Applied Catalysis B: Environmental 2021, 282, 119584.
[85] J. Shi, F. Qiu, W. Yuan, M. Guo, Z. Lu, Chem. Eng. J. 2021, 403, 126312.
[86] A. Chunduri, S. Gupta, O. Bapat, A. Bhide, R. Fernandes, M. K. Patel, V. Bambole, A. Miotello, N. Patel, Applied Catalysis B: Environmental 2019, 259, 118051.
[87] Y. Chen, L. Yang, C. Li, Y. Wu, X. Lv, H. Wang, J. Qu, Energy & Environ Materials 2023, 0, e12590.
[88] Y. Li, H. Xu, P. Yang, R. Li, D. Wang, P. Ren, S. Ji, X. Lu, F. Meng, J. Zhang, M. An, Mater. Today Energy 2022, 25, 100975.
